# Stigmasterol–Acylglycerol Conjugates: Chemoenzymatic Synthesis as a Shorter Alternative to Chemical Routes and the Impact of Fatty Acid Chain Length on the Physicochemical Parameters of Liposomes

**DOI:** 10.3390/molecules31183355

**Published:** 2026-09-21

**Authors:** Witold Gładkowski, Marcin Olesiński, Hubert Fortuna, Małgorzata Serowik, Anna Chojnacka, Aleksandra Włoch

**Affiliations:** 1 Department of Food Chemistry and Biocatalysis, Wrocław University of Environmental and Life Sciences, Norwida 25, 50-375 Wrocław, Poland; hubertfortuna546@gmail.com (H.F.); anna.chojnacka@upwr.edu.pl (A.C.); 2 Department of Physics and Biophysics, Wrocław University of Environmental and Life Sciences, Norwida 25, 50-375 Wrocław, Poland; marcinolesinski@protonmail.com; 3 Institute of Agricultural Engineering, Wrocław University of Environmental and Life Sciences, Chełmońskiego 37A, 51-630 Wrocław, Poland; malgorzata.serowik@upwr.edu.pl

**Keywords:** stigmasterol, acylglycerols, lipases, chemoenzymatic synthesis, liposomes

## Abstract

In this study, a two-step chemoenzymatic route was developed for the synthesis of symmetrical acylglycerol–stigmasterol conjugates bearing a stigmasterol moiety at the *sn*-2 position and saturated acyl chains (decanoyl, C10, or stearoyl, C18) at the *sn*-1,3 positions. The use of *Candida antarctica* lipase B (CALB) under mild conditions (0–5 °C) ensured high regioselectivity, successfully preventing acyl migration and eliminating the need for complex protection/deprotection steps. The impact of the synthesized conjugates on DPPC (1,2-dipalmitoyl-sn-glycero-3-phosphocholine) model lipid membranes was evaluated using dynamic light scattering (DLS), differential scanning calorimetry (DSC), attenuated total reflectance Fourier transform infrared spectroscopy (ATR-FTIR), and fluorescence spectroscopy. Incorporation of the conjugates broadened the main phase transition of DPPC, reduced membrane fluidity in the liquid-crystalline phase, and limited interfacial hydration in a concentration- and chain-length-dependent manner. The short-chain derivative (C10) maintained smaller, homogeneous liposomes and disrupted lipid packing in the gel phase, whereas the longer-chain derivative (C18) promoted stronger hydrophobic interactions within the bilayer core. These findings highlight the potential of chemoenzymatically derived lipid–phytosterol conjugates for the precise tailoring of nanocarriers in functional food and nutraceutical delivery systems.

## 1. Introduction

Phytosterols (PS) are naturally occurring plant sterols that are essential structural components of plant cell membranes, where they contribute to membrane stability and integrity [1,2]. Owing to their diverse biological activities, they have attracted considerable interest as functional food ingredients and bioactive compounds. The best-documented effect of PS is their ability to reduce intestinal cholesterol absorption, leading to decreased low-density lipoprotein cholesterol (LDL-C) levels and a lower risk of cardiovascular disease [3,4,5]. Beyond their cholesterol-lowering properties, phytosterols exhibit anticancer, anti-inflammatory, antioxidant, antidiabetic, and immunomodulatory activities [6,7,8,9,10,11], making them promising agents for the prevention and management of chronic disorders, including obesity, autoimmune diseases, and neurodegenerative conditions [12,13,14,15].

However, free phytosterols suffer from limited solubility in both oil and water, high melting points, susceptibility to oxidation, a chalky taste, and poor bioavailability. These drawbacks have prompted the development of lipid-based derivatives to enhance their delivery to target tissues. Among the various esterified forms of phytosterols, including conjugates with fatty acids [16,17], glycerophospholipids [18], phenolic acids [19,20], or catechins [21], conjugates with acylglycerols are particularly attractive. Acylglycerols are naturally present in foods and the human body. They have been widely employed as lipid-based delivery systems [22,23] for various bioactive compounds, including carotenoids [24], non-steroidal anti-inflammatory drugs [25], or sunscreens of the cinnamate class [26]. In addition to their carrier function, acylglycerols serve as effective building blocks for liposomal formulations, which can further enhance the stability and bioavailability of stigmasterol.

In our previous studies, we reported the synthesis, thermo-oxidative stability, and impact on lipid bilayer properties of acylglycerols containing one or two stigmasterol moieties attached to the glycerol backbone [27,28,29,30]. In particular, symmetrical acylglycerol–stigmasterol conjugates bearing a stigmasterol residue at the internal *sn*-2 position and fatty acid residues at the external *sn*-1 and *sn*-3 positions of the glycerol backbone were synthesized via three-step procedures using 2-benzyloxypropane-1,3-diol or dihydroxyacetone as starting materials. To establish a shorter synthetic route, the present study describes a two-step chemoenzymatic synthesis of 1,3-distearoyl-2-stigmasterylsuccinoylglycerol and 1,3-didecanoyl-2-stigmasterylsuccinoylglycerol. The developed methodology employs a lipase-catalyzed acylation of glycerol, a renewable and readily available bio-based feedstock [31,32,33]. Furthermore, the application of lipase catalysis provides an attractive alternative to conventional chemical steps by enabling selective transformations under mild reaction conditions, thereby reducing energy consumption and minimizing waste generation [34,35].

Liposomal encapsulation of phytosterols is one of the most promising strategies to enhance their stability by protecting them from thermal and oxidative degradation during processing and storage [36,37,38]. In addition, liposomal delivery to target tissues can improve bioavailability of phytosterols, potentially reducing the amount of PS required to achieve a cholesterol-lowering effect. This approach may also limit dietary exposure to oxidized phytosterol derivatives, which form during processing and may exert undesirable biological effects [39].

The aim of this study was to evaluate the influence of aliphatic chain length in novel stigmasterol–acylglycerol conjugates on membrane dynamics. Biophysical studies were conducted on model liposomal bilayers composed of 1,2-dipalmitoyl-*sn*-glycero-3-phosphocholine (DPPC) and newly synthesized symmetrical acylglycerols. Having previously examined the behavior of myristic (C14) and palmitic (C16) derivatives, this study specifically focuses on comparing decanoyl (C10) and stearic (C18) analogs—representing significantly shorter and longer acyl chains—to capture a broader structural spectrum and systematically elucidate how these distinct variations in chain length dictate the physicochemical, structural, and thermotropic properties of these binary lipid systems. These lipid conjugates were prepared via a newly developed, two-step chemoenzymatic route, providing a shorter and more streamlined alternative to conventional chemical methods.

## 2. Results and Discussion

### 2.1. Chemoenzymatic Synthesis of Acylglycerol-Stigmasterol Conjugates ***4a*** and ***4b***

The previously reported synthetic pathways for preparing symmetrical acylglycerols bearing a stigmasterol moiety at the *sn*-2 position involved a three-step synthesis starting from commercially available glycerol derivatives. These routes comprised the esterification of either 2-benzyloxypropane-1,3-diol or dihydroxyacetone with the corresponding fatty acids, followed by benzyl group deprotection or carbonyl reduction, respectively, to afford 1,3-diacylglycerol intermediates. In the final step, the free hydroxyl group at the *sn*-2 position of these intermediates was esterified with stigmasterol derivatives [28,30]. However, both synthetic approaches suffered from significant limitations. In the dihydroxyacetone-based route, acyl migration occurred during carbonyl reduction, whereas in the 2-benzyloxypropane-1,3-diol-based route, conventional hydrogenolytic removal of the benzyl protecting group was incompatible with intermediates containing unsaturated fatty acid residues.

Using the synthetic pathways described above, we previously obtained symmetrical acylglycerols containing myristic, palmitic, or oleic acid residues at the *sn*-1 and *sn*-3 positions and a stigmasterol moiety linked to the *sn*-2 position via a succinyl linker. In the present study, we sought to determine how variations in the fatty acid chain length of these conjugates influence the physicochemical properties of model liposomes. Therefore, we synthesized decanoyl (C10) and stearoyl (C18) analogues, chosen specifically for their marked difference in chain length.

**Scheme 1:**
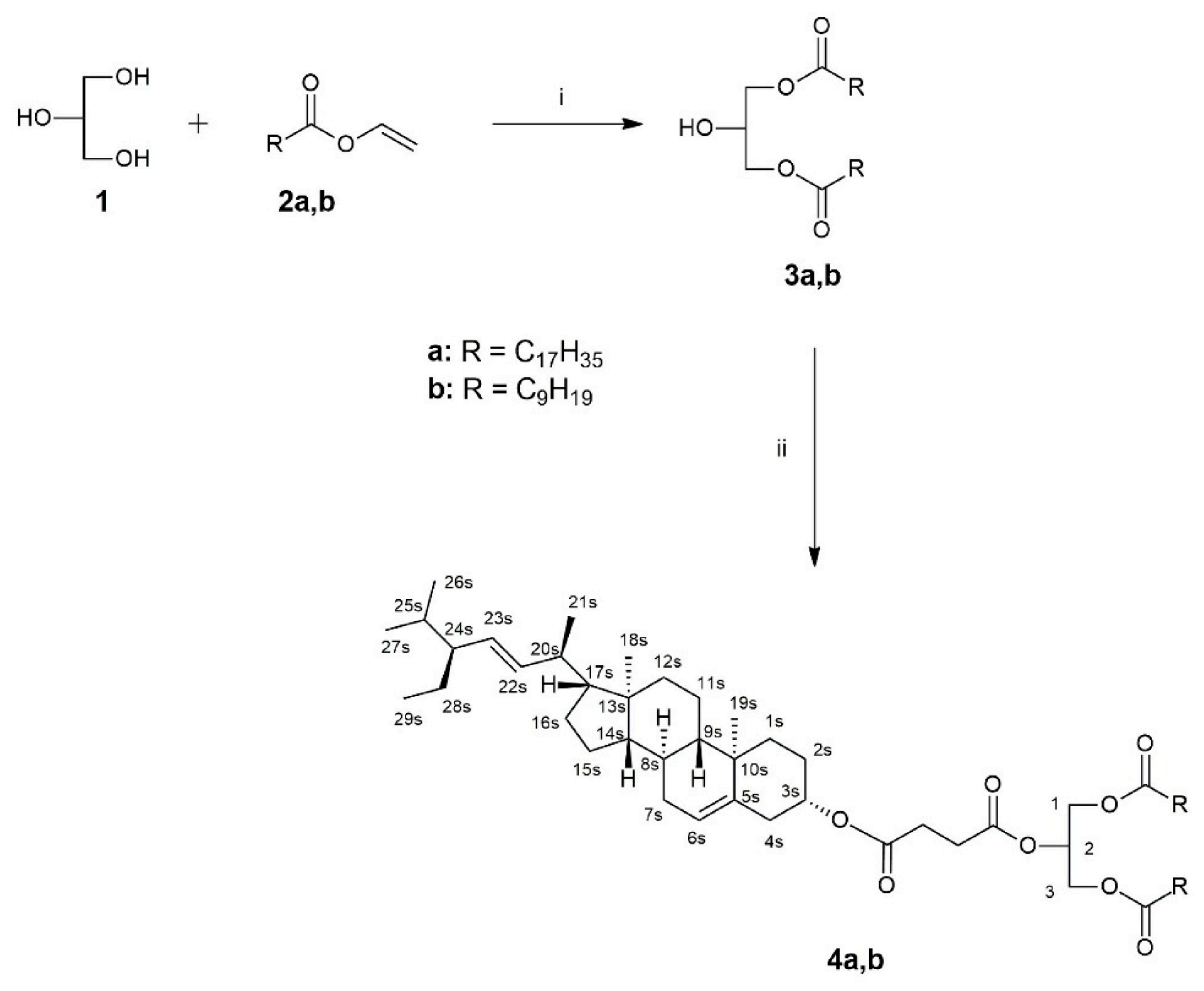
Two-step chemoenzymatic synthesis of acylglycerol–stigmasterol conjugates **4a** and **4b.** *Reagents and conditions:* (i) CALB, anhydrous CH_2_Cl_2_, 0–5°, and 5 h. (ii) Stigmasterol hemisuccinate, DCC, DMAP, CHCl_3_, r.t., and 24 h.

To establish a shorter synthetic strategy as an alternative to previously reported chemical methods, we developed a two-step chemoenzymatic approach starting directly from glycerol (**1**) (Scheme 1).

In the first step, glycerol (**1**) was subjected to enzymatic acylation, following the transesterification procedure described by Halldorsson et al. [40], to regioselectively introduce the corresponding fatty acyl residues at the *sn*-1 and *sn*-3 positions. The reaction, catalyzed by *Candida antarctica* lipase B (CALB) immobilized on an acrylic resin, employed vinyl stearate or vinyl decanoate as the acyl donor and was performed at 0–5 °C for 5 h in dichloromethane. Only the minimum amount of solvent required to dissolve the substrates was used. After purification by flash chromatography, the resulting 1,3-diacylglycerols **3a** and **3b** were coupled with stigmasteryl hemisuccinate via Steglich esterification to afford the target compounds 1,3-distearoyl-2-stigmasterylsuccinoylglycerol (**4a**) and 1,3-didecanoyl-2-stigmasterylsuccinoylglycerol (**4b**).

The yields of 1,3-diacylglycerols **3a** and **3b** were primarily influenced by the regioselectivity of the lipase, potential acyl migration, and competing hydrolytic side reactions. Reaction monitoring by thin-layer chromatography (TLC), in line with observations reported by Halldorsson et al. [40], revealed no detectable formation of 1,2-diacylglycerols during the 5 h reaction period, indicating that acyl migration was effectively suppressed under the applied conditions. In contrast, 1(3)-monoacylglycerols and the corresponding free fatty acids were detected as hydrolysis products arising from both the vinyl esters and the 1,3-diacylglycerols, thereby lowering the overall yields of the desired compounds.

CALB was selected as the bioacatalyst because of its high *sn*-1,3 regioselectivity, which has been successfully exploited in the synthesis of structured triacylglycerols [41,42,43,44]. To suppress acyl migration and preserve the regioselectivity of the enzyme, the reaction temperature was maintained at 0–5 °C throughout the process. Furthermore, the reaction time was kept relatively short to further minimize acyl migration. The use of activated vinyl esters as acyl donors was particularly advantageous, as they undergo rapid and essentially irreversible transesterification due to the spontaneous tautomerization of the liberated vinyl alcohol to acetaldehyde [45]. In addition, vinyl esters are well-recognized substrates for CALB, enabling efficient enzymatic acyl transfer.

**Figure 1:**
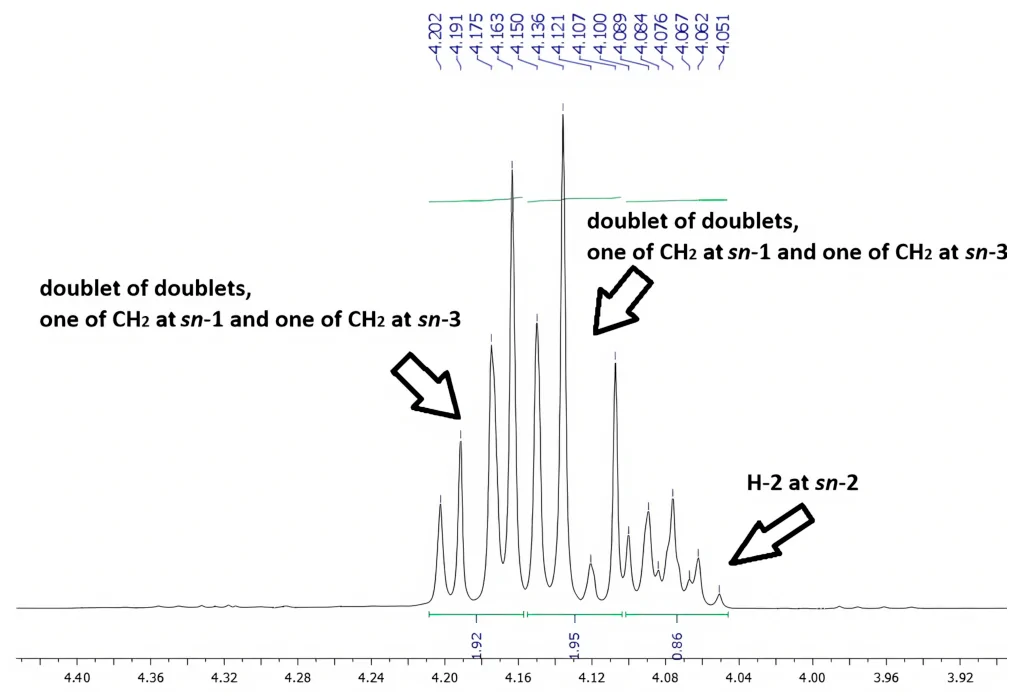
Fragment of ^1^H NMR spectrum of 1,3-distearoylglycerol (**3a**) showing signals corresponding to the glycerol backbone protons.

The structures of the intermediate 1,3-diacylglycerols **3a** and **3b** and the target acylglycerol-stigmasterol conjugates **4a** and **4b** were confirmed by comprehensive ^1^H and ^13^C Nuclear Magnetic Resonance (NMR) spectroscopy, including two-dimensional homonuclear correlation spectroscopy (^1^H-^1^H COSY), ^1^H-^13^C heteronuclear multiple-quantum correlation (HMQC) or heteronuclear single quantum correlation (HSQC), and ^1^H-^13^C heteronuclear multiple-bond correlation (HMBC) experiments (Figures S1–S20, Supplementary Materials). The complete spectroscopic data are provided in Section 3.4 and Section 3.5. As demonstrated in our previous studies [28,30], the regioisomeric purity of 1,3-diacylglycerols can be readily confirmed by their characteristic NMR signals, as illustrated for 1,3-distearoylglycerol (**3a**) (Figure 1).

The signals of the methylene protons at the chemically equivalent *sn*-1 and *sn*-3 positions of the glycerol backbone appeared as two doublets of doublets at δ 4.13 (*J* = 11.3, 5.6 Hz) and 4.18 ppm (*J* = 11.3, 4.2 Hz). These signals arise from the diastereotopic nature of the protons within each CH_2_ group, which renders them magnetically non-equivalent despite the chemical equivalence of the *sn*-1 and *sn*-3 positions. The observation of a single pair of resonances for both terminal methylene groups is consistent with the symmetrical 1,3-diacyl substitution pattern and confirms the absence of detectable amounts of the corresponding 1,2-diacylglycerol regioisomers. Further evidence for the exclusive formation of the 1,3-regioisomers was provided by the ^13^C NMR spectra. The carbonyl carbons of the ester groups at the chemically equivalent *sn*-1 and *sn*-3 positions resonated at the same chemical shift (δ 173.9 ppm) in both 1,3-diacylglycerols (**3a,b**). Likewise, the glycerol C-1 and C-3 carbons gave a single resonance at δ 65.00 ppm, consistent with the symmetrical structure of the 1,3-diacylglycerol framework. An analogous spectroscopic pattern was observed for the target conjugates **4a** and **4b**, indicating that no detectable acyl migration occurred during the Steglich esterification.

### 2.2. Preparation of Liposome Formulations

To evaluate the influence of the synthesized stigmasterol conjugates on membrane properties, DPPC liposomes incorporating 1,3-didecanoyl-2-stigmasterylsuccinoylglycerol (**4b**) or 1,3-distearoyl-2-stigmasterylsuccinoylglycerol (**4a**) across a molar ratio range of 0.01–0.10 were prepared using the thin-film hydration method [28,29,30]. Pure DPPC liposomes were used as a control. The resulting multilamellar vesicles (MLVs) were analyzed by differential scanning calorimetry (DSC), whereas extruded unilamellar vesicles (UVs) were prepared for dynamic light scattering (DLS) and fluorescence spectroscopy analyses. The prepared liposomes were then subjected to biophysical studies. The detailed liposome preparation procedures are provided in Section 3.6.

### 2.3. Biophysical Studies

#### 2.3.1. Dynamic Light Scattering Results

The first stage of the biophysical studies focused on determining the hydrodynamic diameter (Z-average) and polydispersity index (PDI) of the control liposomes and liposomes containing the tested conjugates using DLS. Measurement of these parameters plays a key role in the characterization of lipid nanocarriers; liposome size affects their pharmacokinetic profile, physical stability, and tissue retention, whereas the PDI value allows the degree of population homogeneity to be assessed [46].

**Figure 2:**
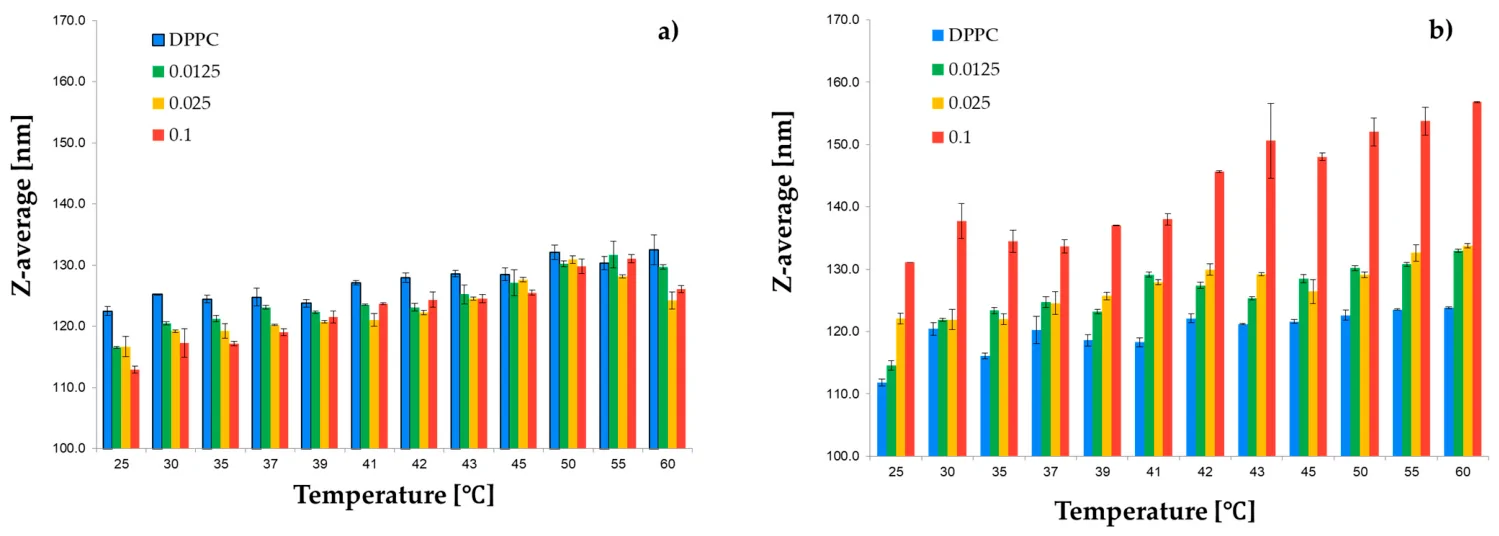
Temperature dependence of the hydrodynamic diameter (Z-average) for the DPPC liposomal matrix (control) and its mixtures with stigmasterol conjugates at various molar ratios: (**a**) 1,3-didecanoyl-2-stigmasterylsuccinoylglycerol (**4b**) and (**b**) 1,3-distearoyl-2-stigmasterylsuccinoylglycerol (**4a**). Data represent mean values ± SD (*n* = 3).

Incorporating the studied conjugates into the DPPC matrix significantly affected the structural parameters of the vesicles, with variations dependent on the acyl-chain length of the modifier, its molar ratio, and temperature. As shown in Figure 2a, liposomes modified with the short-chain (C10) conjugate (**4b**) were consistently smaller across the concentration range (112–120 nm at 25 °C) compared to their corresponding batch-specific pure DPPC control (~122 nm). In contrast, incorporation of the long-chain (C18) conjugate (**4a**) caused a pronounced, concentration-dependent increase in vesicle diameter relative to its batch control (~112 nm at 25 °C), reaching values from 131 nm at 25 °C to 157 nm at 60 °C at the highest molar ratio of 0.1 (Figure 2b). PDI analysis (Table S1) for the C10 derivative (**4b**) showed a high degree of population homogeneity over the entire temperature range, with PDI values generally remaining below 0.13 (minimum values of 0.037–0.049). Moreover, at this maximum molar ratio (0.10), elevated PDI values (~0.15–0.18) were observed at low temperatures (25–30 °C); however, after crossing the main phase-transition region (above 42 °C), the PDI decreased markedly to 0.050–0.076 (Table S2), indicating high population homogeneity in the liquid-crystalline phase [46].

The overall increase in Z-average with increasing temperature observed in all systems may be associated with changes in lipid bilayer properties occurring during the phase transition from the gel state to the liquid-crystalline phase [47]. This transition is accompanied by reduced ordering of the acyl chains, leading to an increase in the area per lipid molecule, increased area compressibility, and enhanced structural fluctuations of the bilayer [48,49]. At the macroscopic level, this results in increased membrane flexibility and vesicle volume, which is directly reflected in the increasing Z-average values. In contrast, the decrease in PDI at higher temperatures—observed particularly in systems with higher modifier contents—indicates a narrowing of the particle-size distribution. This may result from the greater mobility of lipids in the liquid-crystalline phase, which facilitates bilayer reorganization and the formation of a more homogeneous vesicle population.

Most published literature focuses on the effect of chain length on the properties of pure, single-component lipid bilayers [48,49]. In such homogeneous systems, increasing the acyl-chain length strengthens van der Waals interactions, resulting in tighter membrane packing and a decrease in the area per lipid molecule [48]. In contrast, in the binary system investigated in the present study, which contains stigmasterol conjugates as modifiers, liposome size increased with both conjugate chain length and molar ratio. This effect likely does not result from a simple modification of the packing of the DPPC matrix itself, but rather from the geometric and spatial characteristics of the modifier. It may be hypothesized that the presence of the rigid, bulky stigmasterol core in combination with long C18 chains affects local lipid packing and bilayer curvature, thereby promoting the formation of vesicles with a larger hydrodynamic diameter. In contrast, the C10 conjugate exerted a weaker effect in this respect, which is consistent with the smaller vesicle sizes and low PDI values observed.

#### 2.3.2. Results of Differential Scanning Calorimetry (DSC)

**Figure 3:**
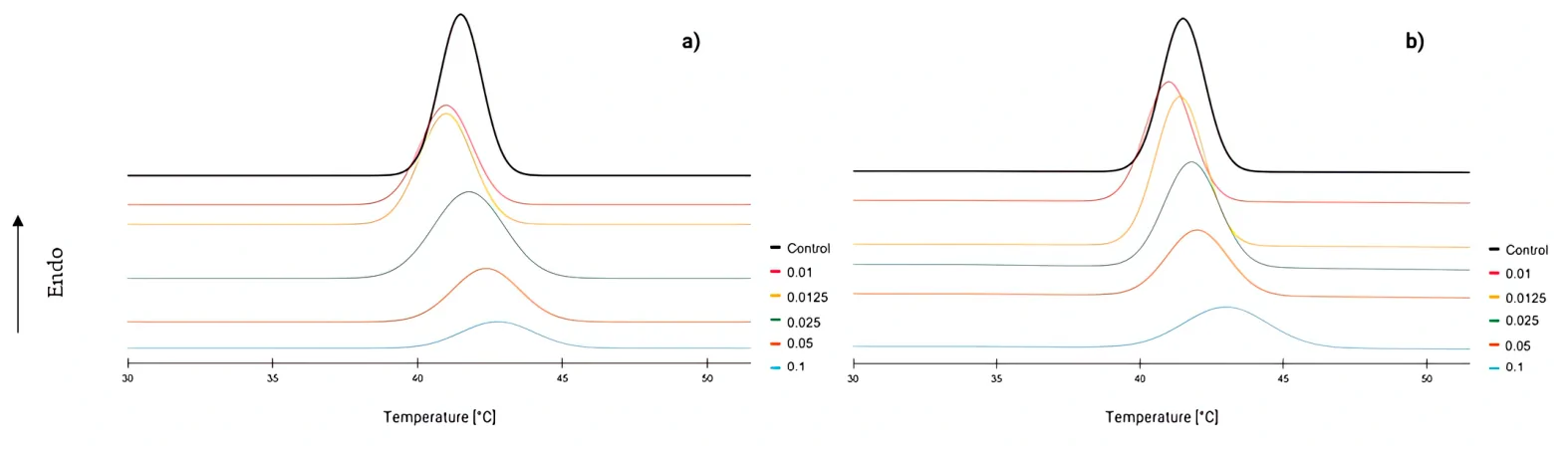
DSC thermograms of DPPC liposomes (control) and liposomes composed of DPPC and the tested conjugate at various molar ratios (n_conjugate_/n_DPPC_): (**a**) conjugate **4b**/DPPC and (**b**) conjugate **4a**/DPPC.

The effects of conjugates **4a** and **4b** incorporated at various conjugate-to-DPPC molar ratios on the thermotropic behavior and main phase transition temperature (T_m_) of model membranes were evaluated by DSC (Figure 3). For pure DPPC, T_m_ was determined to be 41.5 ± 0.2 °C, reflecting a highly cooperative gel-to-liquid-crystalline phase transition that agrees well with literature values [29,50,51]. The incorporation of both conjugates altered the thermotropic profile of the lipid bilayer in a concentration-dependent manner. At low molar ratios (0.01–0.0125), a slight decrease in T_m_ (to ~40.7–41.0 °C) was observed. Increasing the conjugate content to molar ratios of 0.025–0.05 shifted T_m_ toward higher temperatures (~42.0–43.0 °C), accompanied by a gradual peak broadening and loss of symmetry. At the highest molar ratio (0.1), the transition was shifted further toward higher temperatures and was characterized by pronounced peak broadening and increased asymmetry. These changes indicate a reduction in the cooperativity of the gel-to-liquid-crystalline phase transition and increased heterogeneity of lipid organization within the bilayer. Although these effects were observed for both conjugates, peak broadening and flattening were more pronounced for the short-chain C10 derivative **4b** (Figure 3a), whereas the increase in T_m_ was more pronounced for the C18 derivative **4a** (Figure 3b), C18:0. These differences suggest that the effect of the conjugates on DPPC organization results from a balance between packing perturbation caused by the bulky stigmasterol moiety and hydrophobic interactions involving the fatty acyl chains. The shorter C10 residues may produce a greater hydrophobic mismatch with the C16 acyl chains of DPPC, thereby disrupting regular lipid packing and reducing transition cooperativity. In contrast, the longer C18 residues may interact more favorably with the hydrophobic core of the DPPC bilayer, contributing to the observed shift of Tm toward higher temperatures. At higher conjugate contents, the pronounced broadening and asymmetry of the DSC peaks may indicate increased compositional and structural heterogeneity of the bilayer and non-ideal component mixing. The possible formation of conjugate-enriched domains cannot be excluded; however, DSC alone does not provide direct evidence of phase separation.

Compared with analogous conjugates containing two myristoyl residues (C14:0) and a single stigmasterol moiety [28], the compounds obtained in this study allow the effect of acyl chain length (C10:0 vs. C18:0) on the thermotropic behavior of the lipid membrane to be assessed. The C18:0 acyl residues promote stronger hydrophobic interactions within the bilayer core, thereby increasing membrane ordering and shifting T_m_ toward higher values. Conversely, the shorter residues (C10:0) induce a greater chain-length mismatch relative to the palmitoyl chains of DPPC, which may contribute to the disruption of lipid packing and reduced transition cooperativity. Furthermore, compared to previously studied analogues bearing two stigmasterol moieties [29], the conjugates investigated here contain a single stigmasterol residue at the sn-2 position together with two fatty acyl residues at the *sn*-1 and *sn*-3 positions. This molecular architecture may favor interactions of the fatty acyl chains within the hydrophobic core of the DPPC bilayer while allowing the bulky stigmasterol moiety to locally perturb lipid packing, resulting in a balance between increased membrane ordering and reduced transition cooperativity.

#### 2.3.3. Steady-State Fluorescence Spectroscopy Studies

**Figure 4:**
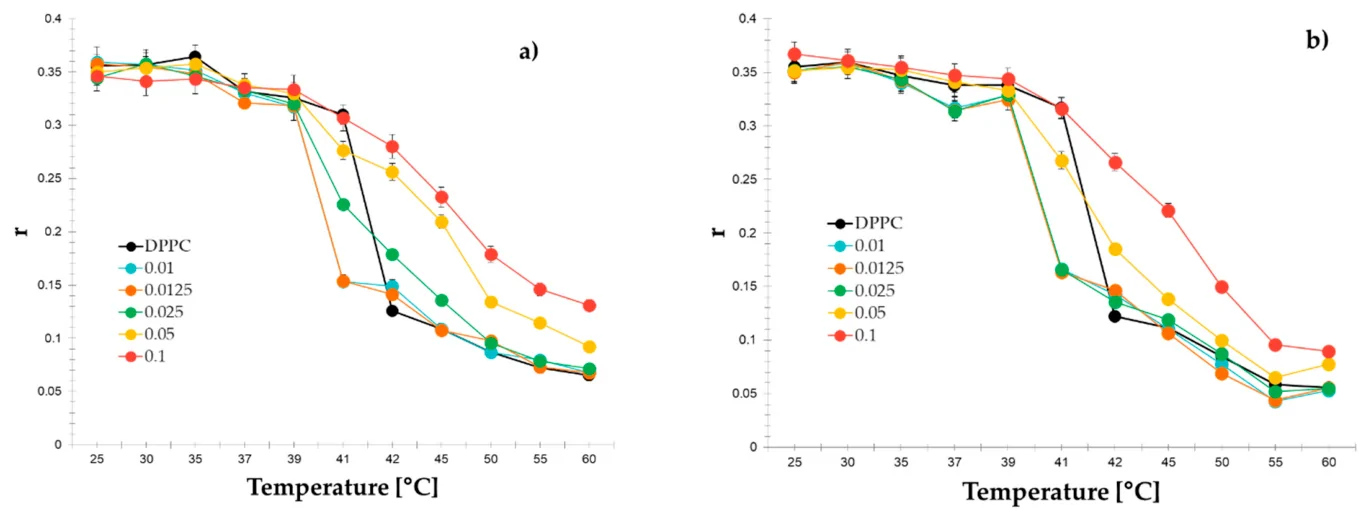
Values of anisotropy of DPH as a function of temperature for DPPC (control) and the different molar ratios of the conjugate to DPPC (n_conjugate_/n_DPPC_) for (**a**) conjugate **4b** and (**b**) conjugate **4a**. Data represent mean values ± SD (*n* = 3).

Fluorescence spectroscopy using the (1,6-diphenyl-1,3,5-hexatriene (DPH) probe was employed to investigate the changes in membrane fluidity and acyl-chain ordering as well as to estimate the main phase transition temperature (T_m_) of the studied lipid systems. Owing to its preferential localization within the hydrophobic region of the lipid bilayer, DPH is sensitive to the rotational mobility and ordering of the hydrocarbon chains, which can be monitored by measuring fluorescence anisotropy (r). Figure 4 shows the temperature dependence of DPH fluorescence anisotropy for pure DPPC liposomes (control) and for DPPC systems containing conjugate **4b** (Figure 4a) or conjugate **4a** (Figure 4b) at various conjugate-to-DPPC molar ratios. For pure DPPC liposomes (control sample), T_m_ was determined to be 41.4 ± 0.4 °C. The gel-to-liquid-crystalline phase transition was sharp and was accompanied by a rapid decrease in fluorescence anisotropy, from r ≈ 0.33 in the gel phase to r ≈ 0.065–0.070 in the liquid-crystalline phase, reflecting the pronounced increase in acyl-chain mobility upon transition. Low conjugate-to-DPPC molar ratios (0.01 and 0.0125) did not significantly affect the fluidity of the bilayer in either phase (values of r were similar to the control), but shifted T_m_ toward lower values (range 39.0–40.0 °C). In the case of the conjugate **4b** at a molar ratio of 0.025, a loss of cooperativity of the process and a broadening of the phase transition temperature range were observed, whereas for conjugate **4a** at the same concentration, the thermotropic response remained similar to that observed at lower molar ratios. At higher molar ratios for both conjugates (0.05 and 0.1), the characteristic sharp decrease in DPH anisotropy associated with the cooperative phase transition of pure DPPC was no longer observed for either conjugate, indicating substantial perturbation of the thermotropic behavior of the bilayer. The addition of conjugate **4b** slightly increased acyl-chain mobility in the gel phase but markedly reduced mobility in the liquid-crystalline phase, as evidenced by the increase in anisotropy to r ≈ 0.131 compared with r ≈ 0.065 for pure DPPC. Conjugate **4a** caused a slight increase in anisotropy in the gel phase and a more pronounced increase in the liquid-crystalline phase. Thus, the C10 conjugate **4b** produced greater changes in acyl-chain organization in the liquid-crystalline phase than the C18 conjugate **4a** at comparable molar ratios. Overall, these results indicate that both conjugates increase acyl-chain ordering and reduce membrane fluidity in the liquid-crystalline phase, particularly at higher conjugate contents.

The fluorescence anisotropy results are generally consistent with the DSC data. At low conjugate-to-DPPC molar ratios (0.01–0.0125), both methods indicated a slight shift of the main phase transition toward lower temperatures. At higher molar ratios, DPH fluorescence revealed a marked loss of the sharp anisotropy drop characteristic of the cooperative DPPC phase transition, whereas DSC showed pronounced broadening and asymmetry of the corresponding thermal transition. Taken together, these observations indicate a substantial reduction in phase-transition cooperativity.

Similar increases in DPH anisotropy in the liquid-crystalline phase have previously been observed for acylglycerol conjugates containing two stigmasterol moieties [29] and for an analogous conjugate containing myristoyl (C14:0) residues and a single stigmasterol moiety [28]. These findings suggest that incorporation of stigmasterol–acylglycerol conjugates into DPPC membranes generally promotes increased ordering and reduced mobility of the acyl chains in the liquid-crystalline phase, although the magnitude of this effect depends on the molecular structure of the conjugate.

To obtain further insight into the effects of conjugates **4a** and **4b** on the membrane interfacial region, fluorescence measurements were performed using 6-dodecanoyl-2-dimethylaminonaphthalene (Laurdan) probe. Laurdan is preferentially located near the glycerol backbone and carbonyl groups of phospholipids at the hydrophilic–hydrophobic interface of the membrane. Owing to this localization, its fluorescence emission is highly sensitive to changes in the polarity and hydration of the surrounding environment associated with lipid phase behavior. In the less hydrated and more ordered gel phase, limited dipolar relaxation of surrounding water molecules results in emission centered at ~440 nm, whereas increased hydration and solvent relaxation in the liquid-crystalline phase shift the emission toward ~490 nm. These spectral changes can be quantified using the generalized polarization (GP) parameter, which provides information on the hydration and organization of the membrane interfacial region.

**Figure 5:**
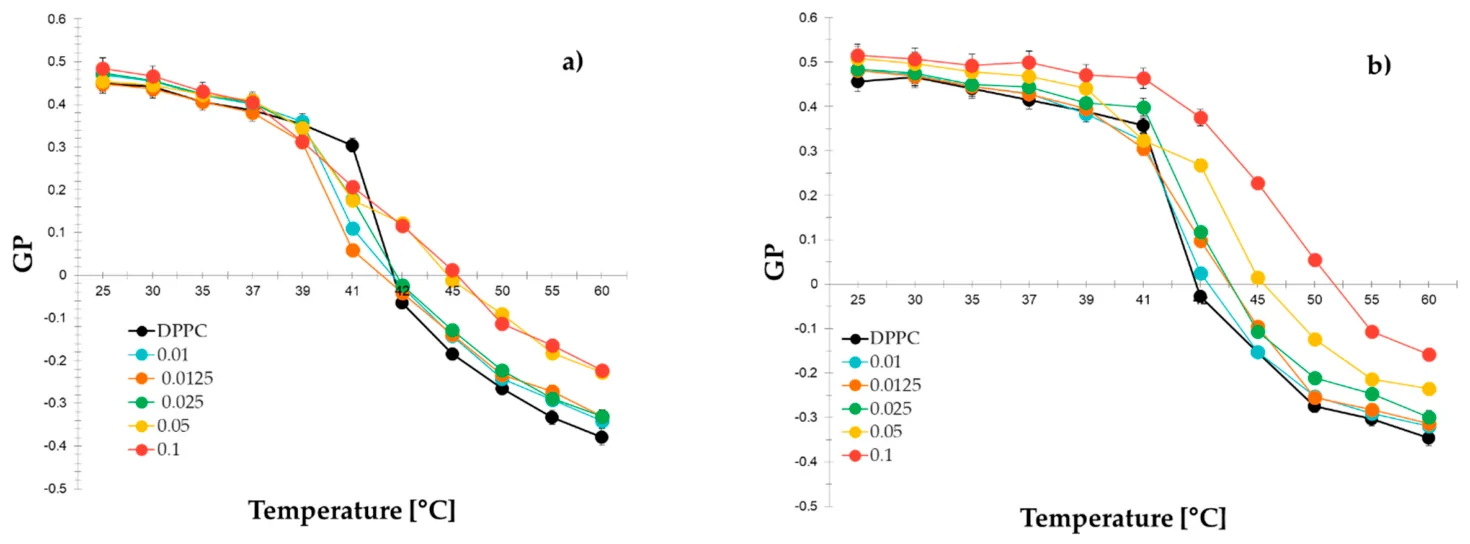
Values of general polarization (GP) of Laurdan as a function of temperature for DPPC (control) and the different molar ratios of the conjugate to DPPC (_nconjugate_/n_DPPC_) for (**a**) conjugate **4b** and (**b**) conjugate **4a**. Data represent mean values ± SD (*n* = 3).

Figure 5 shows the temperature dependence of Laurdan GP for pure DPPC liposomes (control) and for DPPC systems containing conjugate **4b** (Figure 5a) or conjugate **4a** (Figure 5b) at various conjugate-to-DPPC molar ratios. Both compounds affected the organization and hydration of the membrane interfacial region as well as T_m_, although the magnitude and direction of these effects depended on the conjugate structure and molar ratio. Pure DPPC liposomes exhibited a sharp phase transition at ~41.5 °C, accompanied by a pronounced decrease in GP from ~0.45 in the gel phase to −0.38 in the liquid-crystalline phase. This decrease reflects the reduced order and increased hydration of the membrane interfacial region associated with the gel-to-liquid-crystalline phase transition. For the conjugate **4b** (Figure 5a), low conjugate-to-DPPC molar ratios (0.01–0.025) shifted the main phase transition toward lower temperatures and produced a slight increase in GP at higher temperatures, indicating somewhat greater interfacial ordering and/or reduced hydration in the liquid-crystalline phase. At higher molar ratios (0.05 and 0.1), the sharp GP change associated with the DPPC phase transition was substantially broadened over a wider temperature range. These systems also exhibited the highest GP values in the liquid-crystalline phase, indicating increased ordering and reduced hydration of the membrane interfacial region.

Conjugate **4a** (Figure 5b) produced a stronger concentration-dependent increase in GP over the investigated temperature range. At lower molar ratios (0.01–0.025), the effect was most evident in the liquid-crystalline phase, whereas at molar ratios of 0.05 and 0.1, increased GP values were observed in both the gel and liquid-crystalline phases. At the same time, the sharp GP transition characteristic of pure DPPC became progressively broadened. Increasing the content of conjugate **4a** was also associated with a shift of the main phase transition toward higher temperatures. Overall, these results indicate that conjugate **4a** promotes increased ordering and reduced hydration of the membrane interfacial region.

These observations are consistent with previous studies of structurally related acylglycerol–stigmasterol conjugates containing a stigmasterol moiety linked through a succinyl spacer at the *sn*-2 position and different fatty acyl residues at the *sn*-1 and *sn*-3 positions [28,30]. Notably, the conjugate containing myristoyl residues (C14:0), when incorporated at low molar ratios (0.01–0.025), caused a decrease in membrane interfacial ordering near the phase transition and shifted T_m_ toward lower temperatures. This behavior resembles that observed in the present study for the conjugate **4b**. In contrast, the previously studied conjugate containing palmitoyl residues (C16:0)—similarly to the conjugate **4a** investigated here—increased GP values in both the gel and liquid-crystalline phases and shifted T_m_ toward higher temperatures. The previously investigated analogue containing unsaturated oleoyl residues (C18:1) showed distinct behavior, decreasing GP in the gel phase while increasing it in the liquid-crystalline phase. Taken together, these results support the critical role of both fatty acyl chain length and degree of unsaturation in determining the effects of acylglycerol–stigmasterol conjugates on the organization and hydration of lipid bilayers.

**Figure 6:**
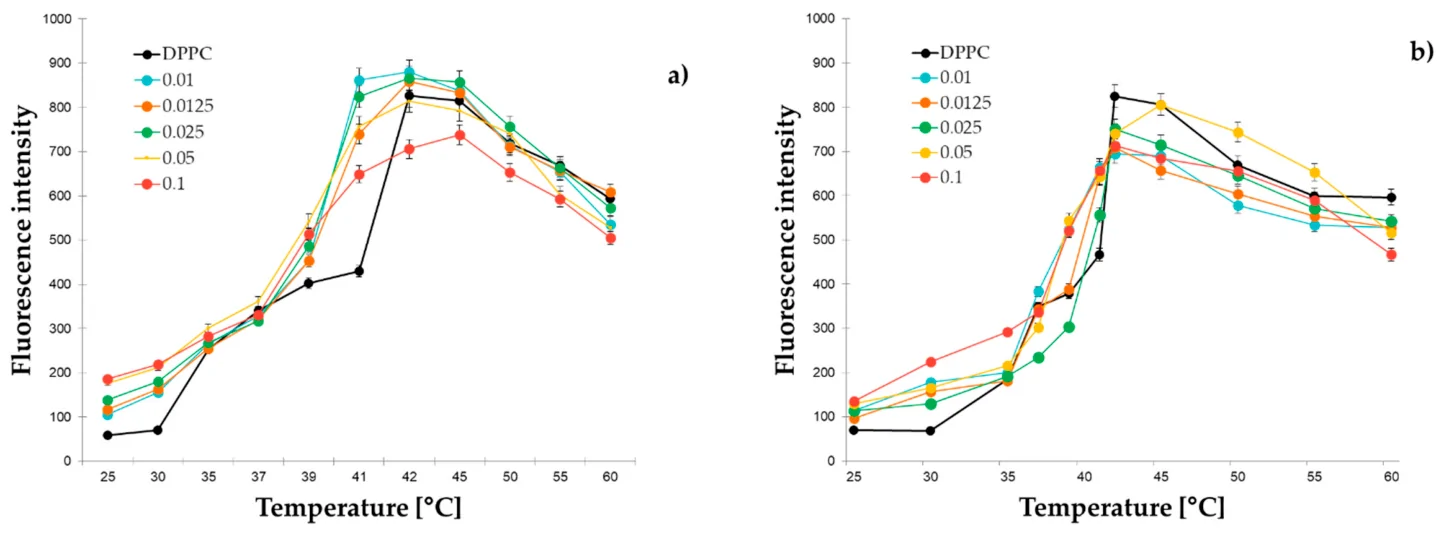
Fluorescence intensity of merocyanine (MC540) as a function of temperature for DPPC (control) and the different molar ratios of the conjugate to DPPC (n_conjugate_/n_DPPC_): (**a**) conjugate **4b** and (**b**) conjugate **4a**. The intensity was measured at the emission maximum at a wavelength of 585 nm. Data represent mean values ± SD (*n* = 3).

To further investigate the effects of conjugates **4a** and **4b** on lipid packing within the membrane interfacial region, fluorescence measurements were performed using merocyanine 540 (MC540). This amphiphilic probe is localized predominantly at the membrane interface—close to the glycerol backbone and polar head group region of phospholipids—and its fluorescence is sensitive to changes in lipid packing and membrane organization. In the tightly packed gel phase, association of MC540 with the membrane is limited, resulting in relatively low fluorescence intensity. During the gel-to-liquid-crystalline phase transition, loosened lipid packing increases the accessibility of the membrane interface to the probe and is generally accompanied by an increase in fluorescence intensity [52,53]. Figure 6 shows the temperature dependence of MC540 fluorescence intensity for pure DPPC liposomes (control) and for DPPC systems containing different molar ratios of conjugate **4b** (Figure 6a) or **4a** (Figure 6b). For pure DPPC, the fluorescence intensity increased markedly in the phase-transition region, reaching a pronounced maximum near the T_m_, followed by a decrease at higher temperatures. Based on these changes, the T_m_ of the control membrane was estimated to be 41.5 ± 0.2 °C, in agreement with literature data [52,54]. As previously reported by Langner and Hui, the fluorescence maximum observed in the phase-transition region reflects not only changes in membrane organization but also the temperature dependence of the fluorescence quantum yield of MC540 [52].

The incorporation of both conjugates altered MC540 fluorescence in both lipid phases, although the magnitude and pattern of these effects depended on the structure and molar ratio of the conjugate. In the gel phase, incorporation of conjugate **4b** caused a concentration-dependent increase in MC540 fluorescence intensity relative to the control, suggesting reduced lipid packing and/or increased accessibility of the membrane interface to the probe. At low conjugate-to-DPPC molar ratios (0.01–0.025), the main phase transition shifted toward lower temperatures, to ~39.5 ± 0.5 °C. In the liquid-crystalline phase, fluorescence intensity remained higher than that of pure DPPC up to ~55 °C, consistent with increased accessibility of the interfacial region to MC540. At elevated temperatures, the fluorescence intensity decreased relative to the control. At the highest molar ratio (0.1), conjugate **4b** produced the largest increase in MC540 fluorescence in the gel phase, indicating the greatest perturbation of lipid packing in the membrane interfacial region. Following the phase transition, the fluorescence profile became broader and the intensity decreased markedly. These observations indicate substantial modification of the membrane environment sensed by MC540. Conjugate **4a** also caused a concentration-dependent increase in MC540 fluorescence in the gel phase, although this effect was less pronounced than that observed for conjugate **4b**. In the liquid-crystalline phase, incorporation of conjugate **4a** resulted in lower fluorescence intensity than that of pure DPPC. Up to ~55 °C, this decrease was more pronounced at the lower molar ratios, whereas at higher temperatures the largest reduction in fluorescence was observed at the highest conjugate content. These results indicate that conjugate **4a** modifies the organization and accessibility of the membrane interfacial region differently from conjugate **4b**.

Overall, the MC540 results indicate that both conjugates perturb lipid organization in the membrane interfacial region. In the gel phase, this perturbation is evidenced by increased MC540 fluorescence, consistent with reduced lipid packing and/or increased probe accessibility, and is more pronounced for the shorter-chain conjugate **4b**. In the liquid-crystalline phase, the lower MC540 fluorescence observed especially for the conjugate **4a** is consistent with reduced accessibility of the membrane interface to the probe. Taken together with the DPH and Laurdan data, these observations support the conclusion that the conjugates modify membrane organization in a concentration- and chain-length-dependent manner.

#### 2.3.4. Results of Attenuated Total Reflectance Fourier Transform Infrared Spectroscopy (ATR-FTIR)

**Figure 7:**
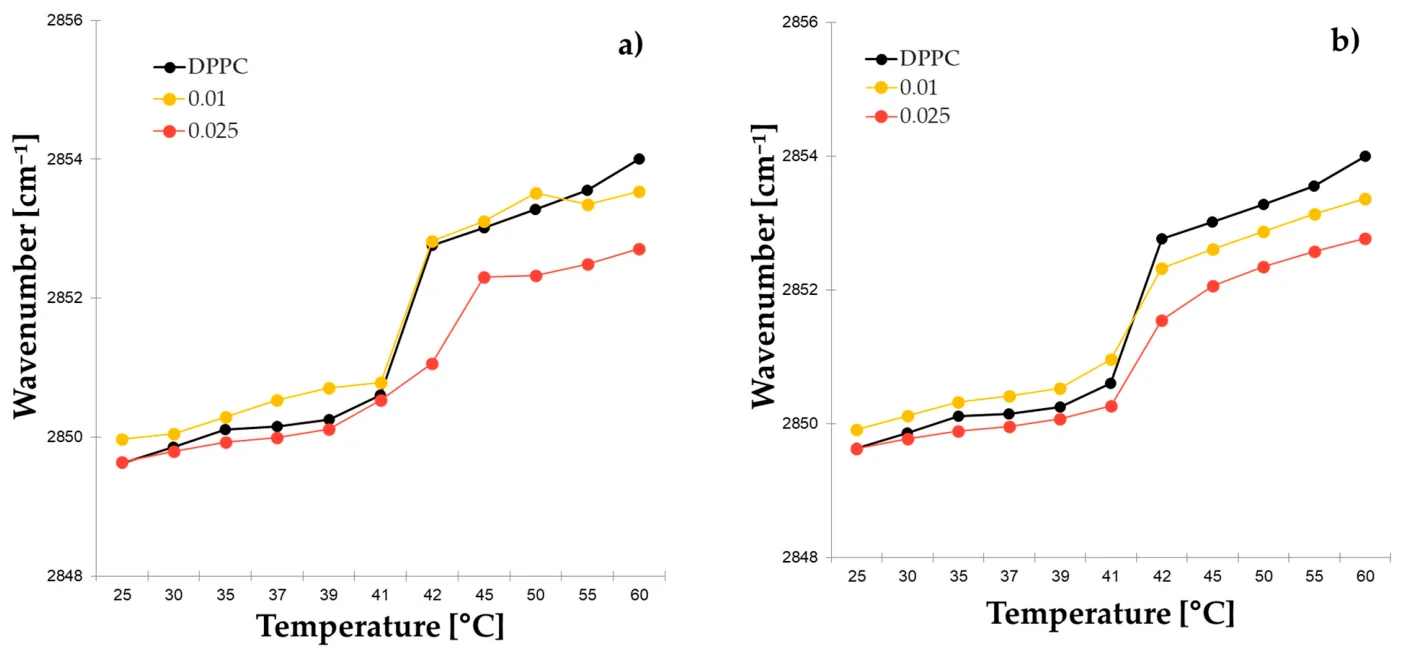
The dependence of the vibration frequency of the asymmetric CH_2_ stretching group on the system temperature for DPPC (control) and different molar ratios of the conjugate to DPPC (n_conjugate_/n_DPPC_) for (**a**) conjugate **4b** and (**b**) conjugate **4a**.

**Figure 8:**
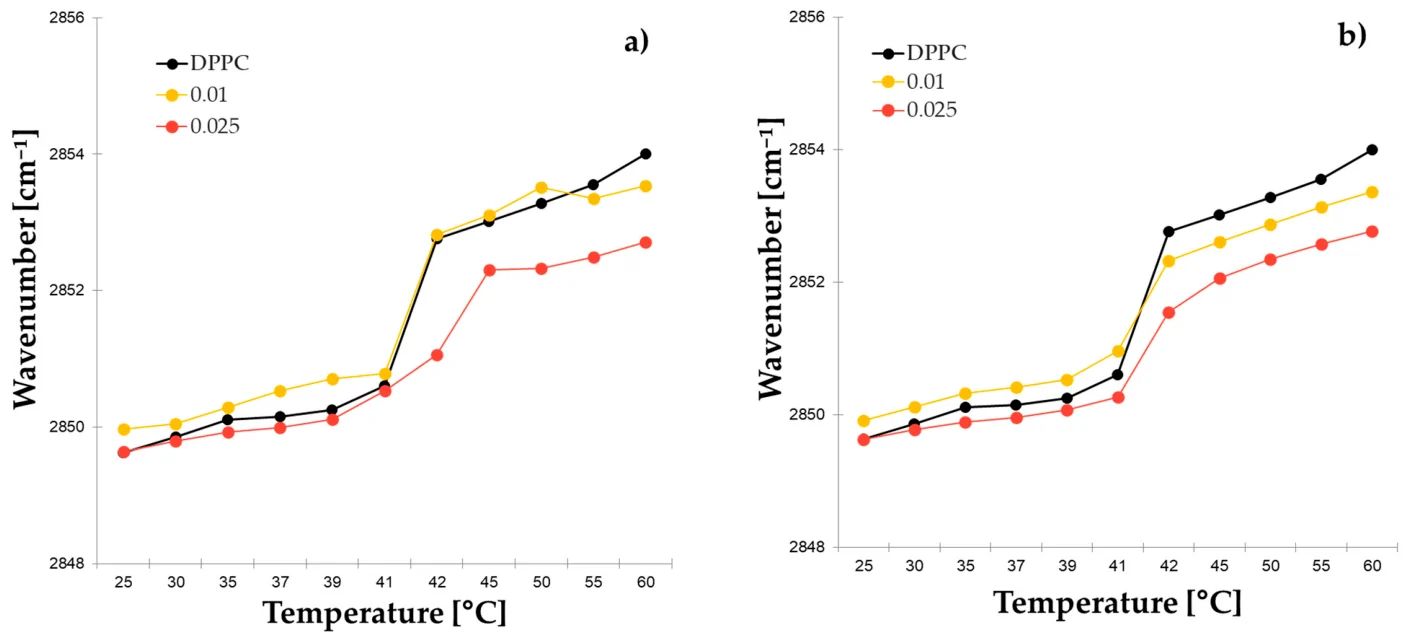
The dependence of the vibration frequency of the symmetric CH_2_ stretching group on the system temperature for DPPC (control) and different molar ratios of the conjugate to DPPC (n_conjugate_/n_DPPC_) for (**a**) conjugate **4b** and (**b**) conjugate **4a**.

To investigate in greater detail the effect of the obtained conjugates on the acyl-chain region at the molecular level, ATR-FTIR spectroscopy was employed. The analysis focused on bands corresponding to methylene stretching vibrations: the asymmetric *ν*_as_(CH_2_) band with a maximum at ~2920 cm^−1^ and the symmetric *ν*_s_(CH_2_) band with a maximum at ~2850 cm^−1^. The frequencies corresponding to the maxima of these bands are closely related to the degree of packing and the dynamics of the lipid bilayer. A shift of the band maxima toward higher frequencies indicates increased conformational freedom and fluidization of the hydrophobic core, whereas a shift toward lower wavenumbers indicates increased ordering and rigidification of the chains [55]. Measurements were performed over a broad temperature range (below and above the phase transition temperature) at two selected conjugate-to-DPPC molar ratios: 0.01 and 0.025. The temperature dependence of the asymmetric and symmetric CH_2_ stretching frequencies for the control sample (DPPC) and DPPC mixtures containing the conjugates is depicted in Figure 7 and Figure 8. The control exhibited the classical curve with a sharp increase in wavenumber across the phase-transition region. From this curve, the phase transition temperature of the control sample was determined to be 41.5 ± 0.2 °C, in agreement with literature data [51] and with the results obtained by DSC and fluorescence spectroscopy.

At the lower molar ratio (0.01), the addition of both conjugates caused fluidization of the acyl-chain region in the gel phase, as indicated by the higher wavenumbers of the asymmetric stretching vibrations compared with pure DPPC (Figure 7a,b; Tables S3 and S4). This behavior is altered in the liquid-crystalline phase (at the two highest temperatures analyzed, 55 and 60 °C), where addition of the shorter-chain conjugate (C10, **4b**) shifted the band maximum toward lower wavenumbers relative to the control. This indicates that at high temperatures, this compound slightly increases the ordering and rigidity of the lipid bilayer without changing the phase transition temperature itself. A similar pattern was observed for the symmetric vibrations (Figure 8a,b; Tables S3 and S4). Addition of the longer-chain conjugate (C18) shifted the maximum of the symmetric stretching band toward lower wavenumbers than those of pure DPPC in the liquid-crystalline phase, likewise indicating a slight increase in ordering. Increasing the concentration of both conjugates to the higher molar ratio (0.025) induced substiantally stronger changes in the system. For both asymmetric and symmetric vibrations, wavenumbers remained lower than those of the control over the entire temperature range, with changes slightly more pronounced for the shorter-chain conjugate (C10, **4b**). This clear decrease in frequency—particularly evident in the fluid phase—unequivocally indicates increased ordering and rigidification of the hydrophobic acyl chains. Moreover, the higher modifier content broadened the sharp phase transition and simultaneously shifted the main transition temperature toward higher values.

The decrease in ν_as_(CH_2_) and ν_s_(CH_2_) stretching frequencies observed by ATR-FTIR in the liquid-crystalline phase at the higher molar ratio (0.025) clearly indicates rigidification of the acyl chains (i.e., reduced bilayer fluidity). These results are consistent with our previous FTIR studies of acylglycerol derivatives containing one or two stigmasterol moieties and palmitic acid, in which addition of the conjugate reduced the fluidity of the hydrophobic region above T_m_ and shifted the band maxima toward lower frequencies [29,30]. Furthermore, the broadening of the phase-transition profile observed by ATR-FTIR and its shift toward higher temperatures at a molar ratio of 0.025 are consistent with the DSC results, which showed reduced cooperativity of the thermotropic phase transition.

## 3. Materials and Methods

### 3.1. Chemicals

Glycerol (**1**, 99.5%), vinyl stearate (**2a**, 95%), vinyl decanoate (**2b**, 95%), lipase B from *Candida antarctica* (CALB, lipase acrylic resin, ≥5000 U/g), *N*,*N*′-dicyclohexylcarbodiimide (DCC, 99%), 4-dimethylaminopyridine (DMAP, ≥99%), ethanol-free chloroform (≥99%), anhydrous dichloromethane (≥98.8%), *n*-hexane (≥99%), ethyl acetate (≥99.5%), and 1,2-dipalmitoyl-*sn*-glycero-3-phosphocholine (DPPC, 99%) were purchased from Sigma-Aldrich (St. Louis, MO, USA). Other chemicals of analytical grade were purchased from Chempur (Piekary Śląskie, Poland). Stigmasteryl hemisuccinate was synthesized from stigmasterol as described previously [27].

### 3.2. Analytical Methods

Analytical TLC was carried out on 0.2 mm aluminum-backed plates precoated with silica gel 60 F254 (Merck, Darmstadt, Germany). The developed chromatograms were visualized either with a staining solution containing 1% Ce(SO_4_)_2_ and 2% H_3_[P(Mo_3_O_10_)_4_] in 10% H_2_SO_4_, followed by heating at 120–200 °C, or by dipping the plates in a 0.05% primuline solution prepared in an acetone/water mixture (4:1, *v*/*v*). Primuline-stained plates were subsequently examined under UV illumination at 365 nm.

NMR spectra, including ^1^H NMR, ^13^C NMR, COSY, HMQC, and HMBC experiments, were recorded using either a JEOL 400 MHz Year Hold Magnet spectrometer (JEOL Ltd., Tokyo, Japan) or a Bruker Avance II 600 MHz spectrometer (Bruker, Rheinstetten, Germany). All samples were prepared in CDCl_3_, and chemical shifts were referenced to the residual solvent signals at δ_H_ 7.26 ppm and δ_C_ 77.00 ppm. Signals arising from the stigmasterol moiety in the data presented in Section 3.5 are designated by the letter “s”.

Melting points of the synthesized compounds were determined using a Boetius apparatus (Nagema, Dresden, Germany).

### 3.3. Purification of Compounds

Purification of the synthesized compounds was performed by flash chromatography using a puriFlash^®^ SX520 Plus Interchim system (Interchim, Montluçon, France) equipped with a UV detector, gradient pump, and fraction collector. Separations were carried out on puriFlash^®^ SIHP-JP F0040 (30 μm) (Interchim, Montluçon, France) columns. Samples were applied by dry loading onto a puriFlash^®^ F0012 (Interchim, Montluçon, France) pre-column, and elution was performed using gradient mixtures of hexane and ethyl acetate at varying ratios. The mobile phase was delivered at a flow rate of 26 mL/min under a pressure of 15 mbar.

### 3.4. Enzymatic Preparation of 1,3-Diacylglycerols ***3a*** and ***3b***

#### General Procedure

To a solution of glycerol **1** (1.0 equiv.) and the corresponding vinyl ester **2** (2.5 equiv.) in anhydrous dichloromethane (10 mL), CALB (50 wt% relative to glycerol) was added. The reaction mixture was stirred magnetically in a 100 mL round-bottom flask in an ice bath at 0–5 °C for 5 h. Upon completion of the reaction, the enzyme was removed by vacuum filtration through a funnel fitted with a sintered glass disc (porosity G4), and the solvent was evaporated under reduced pressure. The crude product was purified by flash chromatography using a gradient elution of *n*-hexane to *n*-hexane/ethyl acetate (5:1, *v*/*v*).

The reaction yields and physical and spectroscopic data for the synthesized 1,3-diacylglycerols are given below:

1,3-distearoylglycerol (**3a**)

Obtained from 0.21 g of glycerol; yield 0.54 g (38%); white crystals; mp 75–77 °C (lit. 73–73.4 °C, [40]; R_f_ = 0.12 (*n*-hexane/ethyl acetate, 5:1, *v*/*v*).

^1^H NMR (400 MHz, CDCl_3_) δ: 0.87 (t, *J* = 6.9 Hz, 6H, 2 × -OC(O)(CH_2_)_16_CH_3_), 1.20–1.34 (m, 56H, 2 × -OC(O)CH_2_CH_2_(CH_2_)_14_CH_3_), 1.58–1.66 (m, 4H, 2 × -OC(O)CH_2_CH_2_(CH_2_)_14_CH_3_), 2.34 (t, *J* = 7.6 Hz, 4H, 2 × -OC(O)CH_2_(CH_2_)_15_CH_3_), 4.08 (m, 1H, H-2 at *sn*-2), 4.13 (dd, *J* = 11.1, 5.5 Hz, 2H, one of CH_2_-1 at *sn*-1, one of CH_2_-3 at *sn*-3), 4.18 (dd, *J* = 11.1, 4.5 Hz, 2H, one of CH_2_-1 at *sn*-1, one of CH_2_-3 at *sn*-3); ^13^C NMR (100 MHz): 14.11 (2 × -OC(O)(CH_2_)_16_CH_3_), 22.68 (2 × -OC(O)(CH_2_)_15_CH_2_CH_3_), 24.88 (2 × -OC(O)CH_2_CH_2_(CH_2_)_14_CH_3_), 29.11, 29.35, 29.44, 29.59, 29.65, 29.69 (2 × -OC(O)CH_2_CH_2_(CH_2_)_12_CH_2_CH_2_CH_3_), 31.91 (2 × -OC(O)(CH_2_)_14_CH_2_CH_2_CH_3_), 34.09 (2 × -C(O)CH_2_(CH_2_)_15_CH_3_), 65.02 (C-1, C-3), 68.37 (C-2), 173.92 (2 × -OC(O)(CH_2_)_16_CH_3_)

1,3-didecanoylglycerol (**3b**)

Obtained from 0.11 g of glycerol; yield 0.24 g (51%); white crystals; mp 35–39 °C (lit. 40.9–41.2 °C, [40]; R_f_ = 0.11 (*n*-hexane/ethyl acetate 5:1, *v*/*v*).

^1^H NMR (400 MHz, CDCl_3_) δ: 0.87 (t, *J*= 6.9 Hz, 6H, 2 × -OC(O)(CH_2_)_8_CH_3_), 1.20–1.36 (m, 24H, 2 × -OC(O)CH_2_CH_2_(CH_2_)_6_CH_3_), 1.58–1.66 (m, 4H, 2 × -OC(O)CH_2_CH_2_(CH_2_)_6_CH_3_), 2.34 (t, *J* = 7.6 Hz, 4H, 2 × -OC(O)CH_2_(CH_2_)_7_CH_3_), 4.08 (m, 1H, H-2 at *sn*-2), 4.13 (dd, *J* = 11.2, 5.6 Hz, 2H, one of CH_2_-1 at *sn*-1, one of CH_2_-3 at *sn*-3), 4.18 (dd, *J* = 11.2, 4.5 Hz, 2H, one of CH_2_-1 at *sn*-1, one of CH_2_-3 at *sn*-3); ^13^C NMR (100 MHz): 14.07 (2 × -OC(O)(CH_2_)_8_CH_3_), 22.63 (2 × -OC(O)(CH_2_)_7_CH_2_CH_3_), 24.86 (2 × -OC(O)CH_2_CH_2_(CH_2_)_6_CH_3_), 29.09, 29.22, 29.38 (2 × -OC(O)CH_2_CH_2_(CH_2_)_4_CH_2_CH_2_CH_3_), 31.82 (2 × -OC(O)(CH_2_)_6_CH_2_CH_2_CH_3_), 34.08 (2 × -OC(O)CH_2_(CH_2_)_7_CH_3_), 65.00 (C-1, C-3), 68.34 (C-2), 173.93 (2 × -OC(O)(CH_2_)_8_CH_3_)

### 3.5. Preparation of Target Acylglycerol-Stigmasterol Conjugates ***4a*** and ***4b***

#### General Procedure

To a solution of 1,3-diacylglycerol **3** (1.0 equiv.), DCC (1.5 equiv.), and DMAP (1.5 equiv.) in 30 mL of ethanol-free chloroform, stigmasteryl hemisuccinate (1.5 equiv.) in chloroform was added dropwise. The reaction mixture was stirred at room temperature until complete consumption of the 1,3-diacylglycerol, as monitored by TLC (24 h; *n*-hexane/ethyl acetate, 5:1, *v*/*v*). The resulting white precipitate was removed by vacuum filtration through a sintered glass disc (porosity G4). The filtrate was washed with 0.5 M HCl, followed by brine until neutral pH was achieved. The organic phase was dried over anhydrous MgSO_4_, filtered, and concentrated under reduced pressure. The crude product was purified by flash column chromatography using a gradient elution of *n*-hexane to *n*-hexane/ethyl acetate (19:1, *v*/*v*).

1,3-Distearoyl-2-stigmasterylsuccinoylglycerol (**4a**)

Obtained from 0.15 g of 1,3-distearoylglycerol (**3a**); yield 0.18 g (68%); white crystals, mp 53–54 °C; R_f_ = 0.36 (*n*-hexane/ethyl acetate, 5:1, *v*/*v*).

^1^H NMR (600 MHz, CDCl_3_): δ 0.69 (s, 3H, CH_3_-18s), 0.79 (d, *J* = 6.5 Hz, 3H, CH_3_-27s), 0.80 (t, *J* = 7.4 Hz, 3H, CH_3_-29s), 0.84 (d, *J* = 6.4 Hz, 3H, CH_3_-26s), 0.87 (t, *J* = 7.0 Hz, 6H, 2 × -OC(O)(CH_2_)_16_CH_3_), 0.95 (td, *J* = 11.6, 5.0 Hz, 1H, H-9s), 1.00 (m, 1H, H-14s), 1.01 (s, 3H, CH_3_-19s), 1.02 (d, *J* = 6.6 Hz, 3H, CH_3_-21s), 1.05 (m, 1H, H-15s (β)), 1.10–1.20 (m, 4H, H-1s (α), H-17s, H-12s (α), one of CH_2_-28s), 1.22–1.32 (m, 57H, 2 × -OC(O)CH_2_CH_2_(CH_2_)_14_CH_3,_ H-16s (β)), 1.38–1.58 (m, 9H, one of CH_2_-28s, H-8s, H-7s (α), CH_2_-11s, H-2s (β), H-24s, H-25s, H-15s (α)), 1.58–1.63 (m, 4H, 2 × -OC(O)CH_2_CH_2_(CH_2_)_14_CH_3_), 1.70 (m, 1H, H-16s (α)), 1.82–1.87 (m, 2H, H-1s (β) and H-2s (α)), 1.93–2.01 (m, 2H, H-7s (β), H-12s (β)), 2.04 (m, 1H, H-20s), 2.29–2.33 (m, 2H, CH_2_-4s), 2.31 (t, *J* = 7.6 Hz, 4H, 2 × -OC(O)CH_2_(CH_2_)_15_CH_3_), 2.58–2.65 (two m, 4H, -OC(O)CH_2_CH_2_(O)CO-Stig), 4.15 (dd, *J* = 12.0, 5.7 Hz, 2H, one of CH_2_-1 at *sn*-1, one of CH_2_-3 at *sn*-3), 4.27–4.31 (two dd, *J* = 12.0, 4.3 Hz, 2H, one of CH_2_-1 at *sn*-1, one of CH_2_-3 at *sn*-3), 4.61 (m, 1H, H-3s), 5.01 (dd, *J* = 15.1, 8.8 Hz, 1H, H-23s), 5.15 (dd, *J* = 15.1, 8.7 Hz, 1H, H-22s), 5.27 (tt, *J* = 5.7, 4.3 Hz, 1H, H-2 at *sn*-2), 5.36 (m, 1H, H-6s); ^13^C NMR (150 MHz, CDCl_3_): δ 12.02 (C-18s), 12.23 (C-29s), 14.10 (2 × -OC(O)(CH_2_)_16_CH_3_), 18.96 (C-27s), 19.28 (C-19s), 20.99 (C-11s), 21.07 (C-26s), 21.20 (C-21s), 22.68 (2 × -OC(O)(CH_2_)_15_CH_2_CH_3_), 24.33 (C-15s), 24.83 (2 × -OC(O)CH_2_CH_2_(CH_2_)_14_CH_3_), 25.39 (C-28s), 27.72 (C-2s), 28.89 (C-16s), 29.13, 29.28, 29.36, 29.50, 29.64, 29.66, 29.70 (2 × -OC(O)CH_2_CH_2_(CH_2_)_12_CH_2_CH_2_CH_3_ and -OC(O)CH_2_CH_2_(O)CO-Stig), 31.83 (C-8s), 31.86 (C-7s and C-25s), 31.92 (2 × -OC(O)(CH_2_)_14_CH_2_CH_2_CH_3_), 34.00 (2 × -OC(O)CH_2_(CH_2_)_15_CH_3_), 36.57 (C-10s), 36.95 (C-1s), 38.04 (C-4s), 39.60 (C-12s), 40.49 (C-20s), 42.18 (C-13s), 50.02 (C-9s), 51.22 (C-24s), 55.91 (C-17s), 56.76 (C-14s), 61.94 (C-1, C-3), 69.44 (C-2), 74.41 (C-3s), 122.72 (C-6s), 129.27 (C-23s), 138.29 (C-22s), 139.50 (C-5s), 171.36 (Stig-OC(O)CH_2_CH_2_(O)CO-), 171.50 (Stig-OC(O)CH_2_CH_2_(O)CO-), 173.31 (2 × -OC(O)(CH_2_)_16_CH_3_)

1,3-Didecanoyl-2-stigmasterylsuccinoylglycerol (**4b**)

Obtained from 0.23 g of 1,3-didecanoylglycerol (**3b**); yield 0.37 g (72%); oily liquid; R_f_ = 0.30 (*n*-hexane/ethyl acetate, 5:1, *v*/*v*).

^1^H NMR (600 MHz, CDCl_3_) δ: 0.69 (s, 3H, CH_3_-18s), 0.79 (d, *J* = 6.5 Hz, 3H, CH_3_-27s), 0.80 (t, *J* = 7.4 Hz, 3H, CH_3_-29s), 0.84 (d, *J* = 6.4 Hz, 3H, CH_3_-26s), 0.87 (t, *J* = 7.0 Hz, 6H, 2 × -OC(O)(CH_2_)_8_CH_3_), 0.94 (td, *J* = 11.6, 5.0 Hz, 1H, H-9s), 1.00 (m, 1H, H-14s), 1.01 (s, 3H, CH_3_-19s), 1.02 (d, *J* = 7.2 Hz, 3H, CH_3_-21s), 1.05 (m, 1H, H-15s (β)), 1.08–1.20 (m, 4H, H-1s (α), H-17s, H-12s (α), one of CH_2_-28s), 1.22–1.32 (m, 25H, 2 × -OC(O)CH_2_CH_2_(CH_2_)_6_CH_3_, H-16s (β)), 1.38–1.57 (m, 9H, one of CH_2_-28s, H-8s, H-7s (α), CH_2_-11s, H-2s (β), H-24s, H-25s, H-15s (α)), 1.58–1.62 (m, 4H, 2 × -OC(O)CH_2_CH_2_(CH_2_)_6_CH_3_), 1.70 (m, 1H, H-16s (α)), 1.82–1.87 (two m, 2H, H-1s (β), H-2s (α)), 1.93–2.00 (m, 2H, H-7s (β), H-12s (β)), 2.04 (m, 1H, H-20s), 2.29–2.33 (m, 2H, CH_2_-4s), 2.31 (t, *J* = 7.6 Hz, 4H, 2 × -OC(O)CH_2_(CH_2_)_7_CH_3_), 2.58–2.65 (two m, 4H, (-OC(O)CH_2_CH_2_(O)CO-Stig), 4.15 (dd, *J* = 12.0, 5.9 Hz, 2H, one of CH_2_-1, one of CH_2_-3), 4.27–4.30 (two dd, *J* = 12.0, 4.3 Hz, 2H, one of CH_2_-1, one of CH_2_-3), 4.60 (m, 1H, H-3s), 5.01 (dd, *J* = 15.1, 8.8 Hz, 1H, H-23s), 5.14 (dd, *J* = 15.1, 8.7 Hz, 1H, H-22s), 5.26 (tt, *J* = 5.9, 4.3 Hz, 1H, H-2), 5.36 (m, 1H, H-6s); ^13^C NMR (150 MHz): δ 12.00 (C-18s), 12.24 (C-29s), 14.10 (2 × -OC(O)(CH_2_)_8_CH_3_), 18.95 (C-27s), 19.28 (C-19s), 20.97 (C-11s), 21.08 (C-26s), 21.19 (C-21s), 22.66 (2 × -OC(O)(CH_2_)_7_CH_2_CH_3_), 24.32 (C-15s), 24.81 (2 × -OC(O)CH_2_CH_2_(CH_2_)_6_CH_3_), 25.39 (C-28s), 27.70 (C-2s), 28.90 (C-16s), 29.09, 29.10, 29.26, 29.27, 29.28, 29.43 (2 × -OC(O)CH_2_CH_2_(CH_2_)_4_CH_2_CH_2_CH_3_, -OC(O)-CH_2_CH_2_-(O)CO-), 31.80 (C-8s), 31.85 (C-7s, C-25s), 31.86 (2 × -OC(O)(CH_2_)_6_CH_2_CH_2_CH_3_), 33.98 (2 × -OC(O)CH_2_(CH_2_)_7_CH_3_), 36.55 (C-10s), 36.92 (C-1s), 38.02 (C-4s), 39.58 (C-12s), 40.49 (C-20s), 42.16 (C-13s), 49.99 (C-9s), 51.20 (C-24s), 55.88 (C-17s), 56.74 (C-14s), 61.93 (C-1, C-3), 69.41 (C-2), 74.39 (C-3s), 122.71 (C-6s), 129.23 (C-23s), 138.30 (C-22s), 139.47 (C-5s), 171.36 (Stig-OC(O)CH_2_CH_2_(O)CO-), 171.51 (Stig-OC(O)CH_2_CH_2_(O)CO-), 173.32 (2 × -OC(O)(CH_2_)_8_CH_3_)

### 3.6. Liposome Preparation

Liposomes were prepared according to previously described methods [28,29,30] with minor modifications. Stock solutions of DPPC and the synthesized stigmasterol conjugates, 1,3-didecanoyl-2-stigmasterylsuccinoylglycerol (**4b**) and 1,3-distearoyl-2-stigmasterylsuccinoylglycerol (**4a**), were prepared in chloroform. Pure DPPC liposomes were used as the control. Lipid mixtures were prepared at conjugate-to-DPPC molar ratios of 0.01, 0.0125, 0.025, 0.05, and 0.10. Chloroform was removed under a gentle stream of nitrogen, and the resulting lipid films were further dried under reduced pressure for at least 1 h to remove residual solvent. The dry lipid films were hydrated with Milli-Q^®^ water above the main phase transition temperature of DPPC (50 °C). The mixture was vortexed mechanically for approximately 15 min until a homogeneous, milky suspension was obtained. Hydration accompanied by mechanical agitation induced the lipid films to detach and self-assemble into multilamellar vesicles (MLVs), which were subsequently used for DSC measurements.

For DLS and fluorescence measurements, unilamellar vesicles (UVs) were prepared by extrusion of the MLV suspensions through polycarbonate membranes with pore sizes of 200 nm and subsequently 100 nm using a Thermobarrel Extruder (10 mL Lipex Extruder, Northern Lipids Inc., Burnaby, BC, Canada) maintained above the main phase transition temperature of DPPC.

### 3.7. Dynamic Light Scattering Analysis

The hydrodynamic diameter (Z-average) and polydispersity index (PDI) of control and conjugate-loaded DPPC liposomes (conjugate-to-DPPC molar ratios of 0.0125, 0.025, and 0.10) were evaluated by DLS using a Zetasizer Nano ZS (Malvern Instruments Ltd., Malvern, UK) at a detection angle of 173°. Liposomal suspensions prepared the previous day (Section 3.6) and stored at 4 °C overnight were allowed to reach room temperature for 1 h prior to testing. To eliminate multiple scattering effects, samples were diluted to a final lipid concentration of 0.1 mg/mL. Data were collected at 25, 30, 35, 37, 39, 41, 42, 43, 45, 50, 55, and 60 °C with a 2 min thermal equilibration time at each temperature step. Results represent three independent experiments (*n* = 3), where each sample was measured in triplicate with 15 runs per measurement.

### 3.8. Differential Scanning Calorimetry Measurements

The thermotropic properties of the liposomal membranes were analyzed using a DSC821e calorimeter (Mettler Toledo, LLC, Columbus, OH, USA) according to previously described methods [28,29], with minor modifications. Multilamellar vesicles (MLVs), obtained after hydration of the lipid films as described in Section 3.6 and prior to extrusion, were used as model membranes. Briefly, following solvent evaporation, the dry lipid films were hydrated with 40 μL of Milli-Q^®^ (Millipore Corporation, Burlington, MA, USA) water and incubated in a thermomixer at 700 rpm for 20 min at a temperature above the main phase transition temperature of DPPC. Then, pure DPPC and DPPC containing conjugates **4a** or **4b** at conjugate-to-DPPC molar ratios of 0.01, 0.0125, 0.025, 0.05, and 0.10 were transferred into 40 μL aluminum pans and hermetically sealed. The final concentration of the lipid in the samples was 25 mg/mL. Before analysis, the samples were stored at 4 °C for 24 h.

Prior to data acquisition, the measurement cell was conditioned for 10 min at 30 °C under a nitrogen atmosphere at a flow rate of 60 mL min^−1^. No additional preheating step was applied to the samples before the actual DSC run. DSC measurements were performed over the temperature range of 25–60 °C at a heating rate of 2 °C min^−1^ under a nitrogen atmosphere at a flow rate of 60 mL min^−1^. An empty sealed aluminum pan was used as the reference. Thermograms were recorded and analyzed using STARe software (version 12.10, Mettler Toledo LLC, Columbus, OH, USA). The main phase transition temperature (T_m_) was determined from the maximum of the main endothermic transition peak. Measurements were performed in three independent experiments (*n* = 3).

### 3.9. Steady-State Fluorescence Spectroscopy Measurements

The main phase transition temperature, hydrophobic core fluidity, hydration and organization of the membrane interfacial region, and lipid packing of DPPC unilamellar vesicles were evaluated using fluorescence spectroscopy according to previously described methods [28,29,54]. Unilamellar vesicles (UVs) prepared as described in Section 3.6 were incubated for 30 min in the dark at room temperature with one of three fluorescent probes: 1,6-diphenyl-1,3,5-hexatriene (DPH), 6-dodecanoyl-2-dimethylaminonaphthalene (Laurdan), or merocyanine 540 (MC540). These fluorescent probes were chosen because of their various affinity to the different areas of the membranes. The final concentration of each fluorescent probe was 1 μM. Measurements were performed for pure DPPC liposomes and DPPC liposomes containing the conjugates at conjugate-to-DPPC molar ratios of 0.01, 0.0125, 0.025, 0.05, and 0.10. The concentration of the lipid in the samples was 0.1 mg/mL.

Fluorescence measurements were performed using a Cary Eclipse spectrofluorometer (Varian, San Diego, CA, USA) equipped with a Peltier temperature controller (accuracy ±0.1 °C) over the temperature range of 25–60 °C in three independent experiments (*n* = 3).

Membrane core fluidity was evaluated using DPH fluorescence anisotropy (r), calculated according to Lakowicz (2006) [56]:

$$r\;=\;\;\frac{I_{VV\;}-{GI}_{VH}}{I_{VV}+{\;2GI}_{VH}}$$

where I represents the intensity; V and H designate the vertical and horizontal orientations of excitation/emission polarizers; and G is the instrumental correction factor calculated as:

$$G\;=\;\frac{I_{HV}}{I_{HH}}$$

The excitation and emission wavelengths for DPH were set to 360 nm and 425 nm, respectively. For MC540, the excitation wavelength was 540 nm, and emission spectra were recorded over the range of 560–650 nm.

Laurdan generalized polarization (GP) was calculated using the following formula [57]:

$$\mathrm{GP}\;=\;\frac{I_{440}\;-\;I_{490}}{I_{440}+\;I_{490}}$$

where I_440_ and I_490_ represent fluorescence intensities measured at emission wavelengths of 440 nm and 490 nm, respectively. Laurdan was excited at 360 nm, and emission spectra were recorded over the range of 400–600 nm.

### 3.10. ATR-FTIR Measurements

ATR-FTIR spectra were recorded using a Nicolet 6700 FTIR spectrometer (Thermo Fisher Scientific, Waltham, MA, USA) equipped with a zinc selenide (ZnSe) ATR accessory. The concentration of the lipid in the samples was 0.1 mg/mL. Based on preliminary experiments, conjugate-to-DPPC molar ratios of 0.01 and 0.025 were selected for analysis, with pure DPPC used as the control.

Measurements were performed according to previously described methods [29,54]. Liposome suspensions prepared as described in Section 3.6 were applied onto the ZnSe crystal and allowed to dry for 1 h under reduced pressure to form a lipid film. After recording spectra of the dry films, the samples were hydrated with 40 μL of Milli-Q^®^ water and incubated above the main phase transition temperature of DPPC for 0.5 h. Spectra were collected over the range of 4000–700 cm^−1^ at a spectral resolution of 2 cm^−1^, with 128 scans co-added for each spectrum. Measurements were performed at 25, 30, 35, 37, 39, 41, 42, 43, 45, 50, 55, and 60 °C in three independent experiments (*n* = 3). Background spectra of Milli-Q^®^ water were recorded under the same conditions and subtracted from the spectra of hydrated samples to minimize the contribution of water absorption. The positions of the bands assigned to symmetric and asymmetric CH_2_ stretching vibrations in the hydrocarbon-chain region were analyzed using OMNIC software (version 8.0, Thermo Fisher Scientific Inc., Waltham, MA, USA).

## 4. Conclusions

In this study, a novel, shorter two-step chemoenzymatic route was successfully developed for the synthesis of symmetrical acylglycerol–stigmasterol conjugates. These compounds bear either stearoyl (C18:0, **4a**) or decanoyl (C10:0, **4b**) chains at the *sn*-1 and *sn*-3 positions, along with a stigmasteryl moiety linked via a succinyl linker at the *sn*-2 position. The application of *Candida antarctica* lipase B (CALB) enabled high regioselectivity under mild reaction conditions, completely eliminating undesirable acyl migration and avoiding cumbersome protection-deprotection strategies. Biophysical evaluation of DPPC model membranes revealed that the conjugates strongly influence bilayer structure, thermotropic behavior, and fluidity in a concentration- and chain-length-dependent manner. The incorporation of both conjugates suppressed the cooperative phase transition of DPPC, reduced membrane fluidity in the liquid-crystalline phase, and limited interfacial hydration. While the shorter C10 chain maintained smaller liposome sizes and caused greater packing disruption in the gel phase, the longer C18 chain promoted stronger hydrophobic interactions in the bilayer core.

Overall, the streamlined chemoenzymatic strategy and the resulting biophysical insights provide a rational framework for designing customized lipid–phytosterol nanocarriers. These findings establish a strong basis for future work exploring such lipid systems across the food and pharmaceutical sectors, where they could simultaneously stabilize liposomal membranes and serve as effective delivery vehicles for bioactive stigmasterol. To build upon these findings, future studies will focus on expanding this conjugate library by synthesizing a series of derivatives featuring shorter or branched acyl chains to systematically tune membrane physical parameters, encapsulation capabilities, and overall nanocarrier stability.

The biophysical evaluations were conducted using single-component model membranes (pure DPPC liposomes). While DPPC serves as a robust and well-characterized model for lipid–phytosterol interaction studies, real biological membranes and complex drug delivery formulations consist of multi-component lipid matrices (e.g., unsaturated lipids, cholesterol, charged lipids) that may alter conjugate behavior. Therefore, future studies need to be expanded to include more complex, multi-component lipid membrane models. Furthermore, once the most promising conjugates are identified, comprehensive in vitro and in vivo evaluations will be conducted to assess their delivery efficiency, safety profile, and bioavailability.

## Data Availability

The data presented in this study are available in the Supplementary Materials of this article.

## Supplementary Materials

The following supporting information can be downloaded at https://www.mdpi.com/article/10.3390/molecules31183355/s1, Figure S1: ^1^H NMR spectrum of 1,3-distearoylglycerol (**3a**); Figure S2: ^13^C NMR spectrum of 1,3-distearoylglycerol (**3a**); Figure S3: DEPT 135 spectrum of 1,3-distearoylglycerol (**3a**); Figure S4: COSY spectrum of 1,3-distearoylglycerol (**3a**); Figure S5: HMQC spectrum of 1,3-distearoylglycerol (**3a**); Figure S6: HMBC spectrum of 1,3-distearoylglycerol (**3a**); Figure S7: ^1^H NMR spectrum of 1,3-didecanoylglycerol (**3b**); Figure S8: ^13^C NMR spectrum of 1,3-didecanoylglycerol (**3b**); Figure S9: DEPT 135 spectrum of 1,3-didecanoylglycerol (**3b**); Figure S10: COSY spectrum of 1,3-didecanoylglycerol (**3b**); Figure S11: HMQC spectrum of 1,3-didecanoylglycerol (**3b**); Figure S12: HMBC spectrum of 1,3-didecanoylglycerol (**3b**); Figure S13: ^1^H NMR spectrum of 1,3-distearoyl-2-stigmasterylsuccinoylglycerol (**4a**); Figure S14: ^13^C NMR spectrum of 1,3-distearoyl-2-stigmasterylsuccinoylglycerol (**4a**); Figure S15: DEPT 135 spectrum of 1,3-distearoyl-2-stigmasterylsuccinoylglycerol (**4a**); Figure S16: COSY spectrum of 1,3-distearoyl-2-stigmasterylsuccinoylglycerol (**4a**); Figure S17: HSQC spectrum of 1,3-distearoyl-2-stigmasterylsuccinoylglycerol (**4a**); Figure S18: HMBC spectrum of 1,3-distearoyl-2-stigmasterylsuccinoylglycerol (**4a**); Figure S19: ^1^H NMR spectrum of 1,3-didecanoyl-2-stigmasterylsuccinoylglycerol (**4b**); Figure S20: ^13^C NMR spectrum of 1,3-didecanoyl-2-stigmasterylsuccinoylglycerol (**4b**); Figure S21: DEPT 135 spectrum of 1,3-didecanoyl-2-stigmasterylsuccinoylglycerol (**4b**); Figure S22: COSY spectrum of 1,3-didecanoyl-2-stigmasterylsuccinoylglycerol (**4b**); Figure S23: HMQC spectrum of 1,3-didecanoyl-2-stigmasterylsuccinoylglycerol (**4b**); Figure S24: HMBC spectrum of 1,3-didecanoyl-2-stigmasterylsuccinoylglycerol (**4b**); Table S1: Temperature dependence of average polydispersity index (PDI) for control (pure DPPC) and systems containing mixtures 1,3-didecanoyl-2-stigmasterylsuccinoylglycerol (**4b**) and DPPC at different molar ratio of (n_acylglycerol_/n_DPPC_): 0.1, 0.025, and 0.0125; Table S2: Temperature dependence of the average polydispersity index (PDI) for pure DPPC and 1,3-distearoyl-2-stigmasterylsuccinoylglycerol *(***4a***)*/DPPC mixtures at molar ratio of acylglycerol to DPPC (n_acylglycerol_/n_DPPC_): 0.1, 0.025, and 0.0125; Table S3: Maxima of asymmetric and symmetric methylene (CH_2_) stretching vibration bands for pure DPPC and 1,3-didecanoyl-2-stigmasterylsuccinoylglycerol (**4b**)/DPPC of acylglycerol to DPPC (n_acylglycerol_/n_DPPC_): 0.01 and 0.025 molar ratios at temperatures; Table S4: Maxima of asymmetric and symmetric methylene (CH_2_) stretching vibration bands for pure DPPC and 1,3-distearoyl-2-stigmasterylsuccinoylglycerol (**4a**)/DPPC mixtures of acylglycerol to DPPC (n_acylglycerol_/n_DPPC_): 0.01 and 0.025 molar ratios at temperatures.
