# Supplementary material for: Stigmasterol–Acylglycerol Conjugates: Chemoenzymatic Synthesis as a Shorter Alternative to Chemical Routes and the Impact of Fatty Acid Chain Length on the Physicochemical Parameters of Liposomes
Source: Molecules. 2026 Sep 21;31(18):3355. doi: 10.3390/molecules31183355 (PMC13609658; doi:10.3390/molecules31183355)
Supplement: Supplementary file 1 [file molecules-31-03355-s001.zip › molecules-4563626-supplementary.pdf]

Supplementary Materials

Stigmasterol-Acylglycerol Conjugates: Chemoenzymatic Syn-thesis as a Shorter Alternative to Chemical Routes and the Impact of Fatty Acid Chain Length on the Physicochemical Parameters of Liposomes

Witold Gładkowski[1*], Marcin Olesiński[2], Hubert Fortuna[1], Małgorzata Serowik[3], Anna Chojnacka[1], Aleksandra Włoch[2*]

[1] Department of Food Chemistry and Biocatalysis, Wrocław University of Environmental and Life Sciences, Norwida 25, 50-375 Wrocław, Poland; anna.chojnacka@upwr.edu.pl (A.C.); hubertfortuna546@gmail.com (H.F.)
[2] Department of Physics and Biophysics, Wrocław University of Environmental and Life Sciences, Norwida 25, 50-375 Wrocław, Poland; marcinolesinski@protonmail.com (M.O.)
[3] Institute of Agricultural Engineering, Wrocław University of Environmental and Life Sciences, Chełmońskiego 37A; 51-630 Wrocław, Poland; malgorzata.serowik@upwr.edu.pl (M.S.)
* Correspondence: witold.gladkowski@upwr.edu.pl (W.G.); aleksandra.wloch@upwr.edu.pl (A.W.)

Table of content

		Page
**Fig. S1**	^{1}H NMR spectrum of 1,3-distearoylglycerol (**3a**)	3
**Fig. S2**	^{13}C NMR spectrum of 1,3-distearoylglycerol (**3a**)	3
**Fig. S3**	DEPT 135 spectrum of 1,3-distearoylglycerol (**3a**)	4
**Fig. S4**	COSY spectrum of 1,3-distearoylglycerol (**3a**)	4
**Fig. S5**	HMQC spectrum of 1,3-distearoylglycerol (**3a**)	5
**Fig. S6**	HMBC spectrum of 1,3-distearoylglycerol (**3a**)	5
**Fig. S7**	^{1}H NMR spectrum of 1,3-didecanoylglycerol (**3b**)	6
**Fig. S8**	^{13}C NMR spectrum of 1,3-didecanoylglycerol (**3b**)	6
**Fig. S9**	DEPT 135 spectrum of 1,3-didecanoylglycerol (**3b**)	7
**Fig. S10**	COSY spectrum of 1,3-didecanoylglycerol (**3b**)	7
**Fig. S11**	HMQC spectrum of 1,3-didecanoylglycerol (**3b**)	8
**Fig. S12**	HMBC spectrum of 1,3-didecanoylglycerol (**3b**)	8
**Fig. S13**	^{1}H NMR spectrum of 1,3-distearoyl-2-stigmasterylsuccinoylglycerol (**4a**)	9

**Fig. S14**	^{13}C NMR spectrum of 1,3-distearoyl-2-stigmasterylsuccinoylglycerol (**4a**)	9
**Fig. S15**	DEPT 135 spectrum of 1,3-distearoyl-2-stigmasterylsuccinoylglycerol (**4a**)	10
**Fig. S16**	COSY spectrum of 1,3-distearoyl-2-stigmasterylsuccinoylglycerol (**4a**)	10
**Fig. S17**	HSQC spectrum of 1,3-distearoyl-2-stigmasterylsuccinoylglycerol (**4a**)	11
**Fig. S18**	HMBC spectrum of 1,3-distearoyl-2-stigmasterylsuccinoylglycerol (**4a**)	11
**Fig. S19**	^{1}H NMR spectrum of 1,3-didecanoyl-2-stigmasterylsuccinoylglycerol (**4b**)	12
**Fig. S20**	^{13}C NMR spectrum of 1,3-didecanoyl-2-stigmasterylsuccinoylglycerol (**4b**)	12
**Fig. S21**	DEPT 135 spectrum of 1,3-didecanoyl-2-stigmasterylsuccinoylglycerol (**4b**)	13
**Fig. S22**	COSY spectrum of 1,3-didecanoyl-2-stigmasterylsuccinoylglycerol (**4b**)	13
**Fig. S23**	HMQC spectrum of 1,3-didecanoyl-2-stigmasterylsuccinoylglycerol (**4b**)	14
**Fig. S24**	HMBC spectrum of 1,3-didecanoyl-2-stigmasterylsuccinoylglycerol (**4b**)	14
**Tab. S1**	Temperature dependence of average polydispersity index (PDI) for control (pure DPPC) and systems containing mixtures 1,3-didecanoyl-2-stigmasterylsuccinoylglycerol (**4b**) and DPPC at different molar ratio of ($n_{acylglycerol}/n_{DPPC}$): 0.1, 0.025, and 0.0125. Values are presented as mean ± standard deviation (*n* = 3).	15
**Tab. S2**	Temperature dependence of the average polydispersity index (PDI) for pure DPPC and 1,3-distearoyl-2-stigmasterylsuccinoylglycerol (**4a**)/DPPC mixtures at molar ratio of acylglycerol to DPPC ($n_{acylglycerol}/n_{DPPC}$): 0.1, 0.025, and 0.0125. Values are presented as mean ± standard deviation (*n* = 3).	15
**Tab. S3**	Maxima of asymmetric and symmetric methylene (CH_2) stretching vibration bands for pure DPPC and 1,3-didecanoyl-2-stigmasterylsuccinoylglycerol (**4b**)/DPPC of acylglycerol to DPPC ($n_{acylglycerol}/n_{DPPC}$): 0.01 and 0.025 molar ratios at temperatures.	16
**Tab. S4**	Maxima of asymmetric and symmetric methylene (CH_2) stretching vibration bands for pure DPPC and 1,3-distearoyl-2-stigmasterylsuccinoylglycerol (**4a**)/DPPC mixtures of acylglycerol to DPPC ($n_{acylglycerol}/n_{DPPC}$): 0.01 and 0.025 molar ratios at temperatures.	16

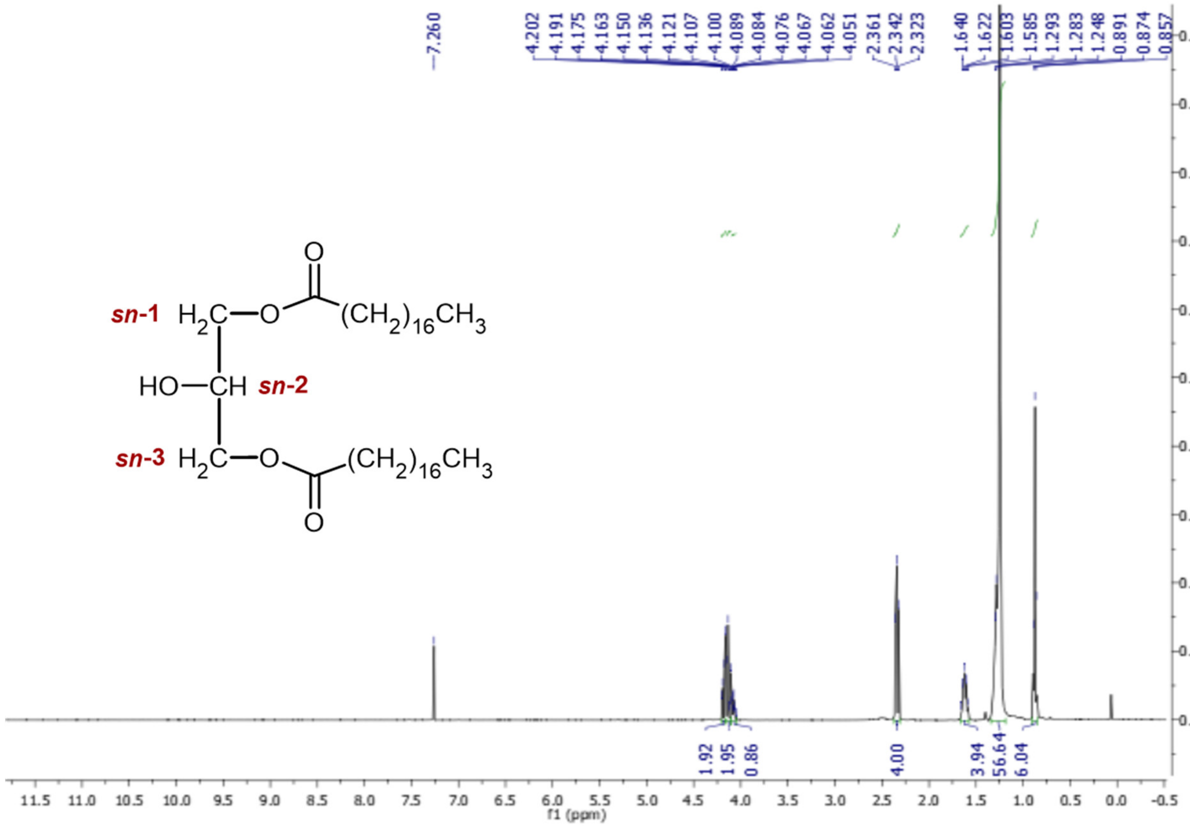

Figure S1. ^{1}H NMR spectrum of 1,3-distearoylglycerol (**3a**)

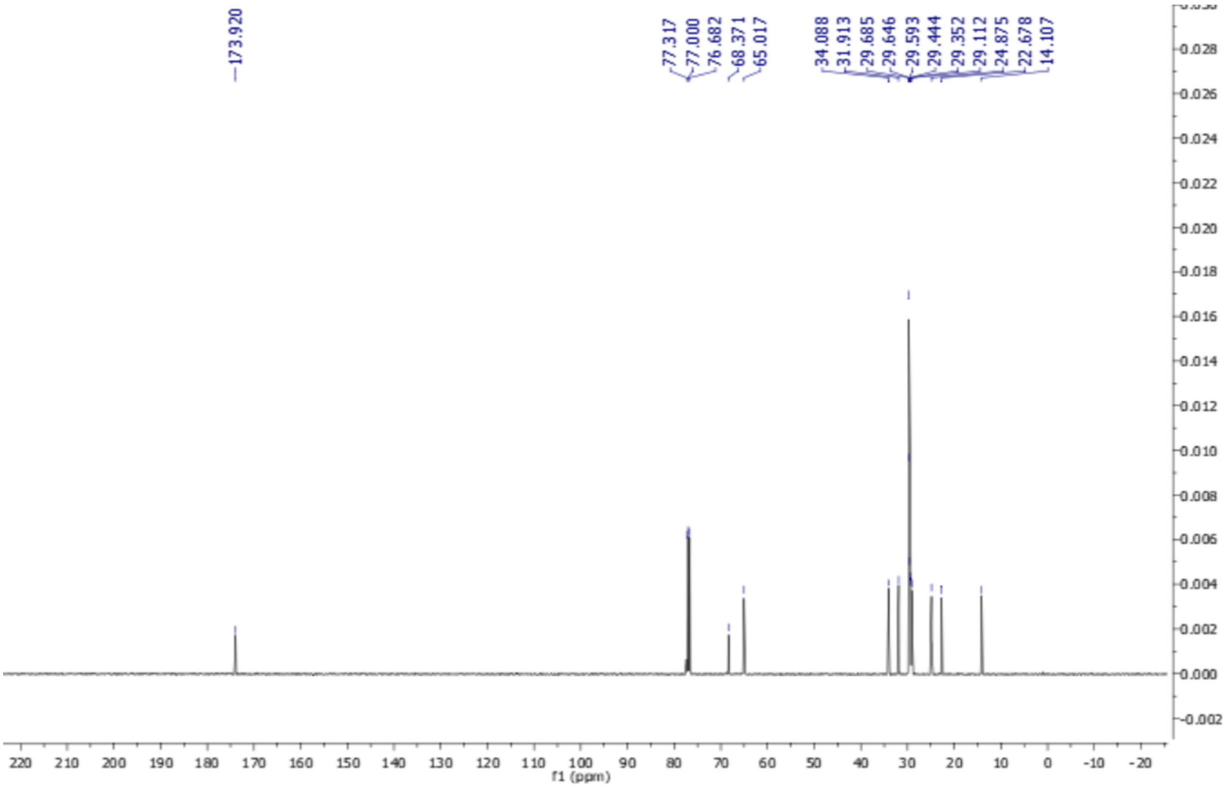

Figure S2. ^{13}C NMR spectrum of 1,3-distearoylglycerol (**3a**)

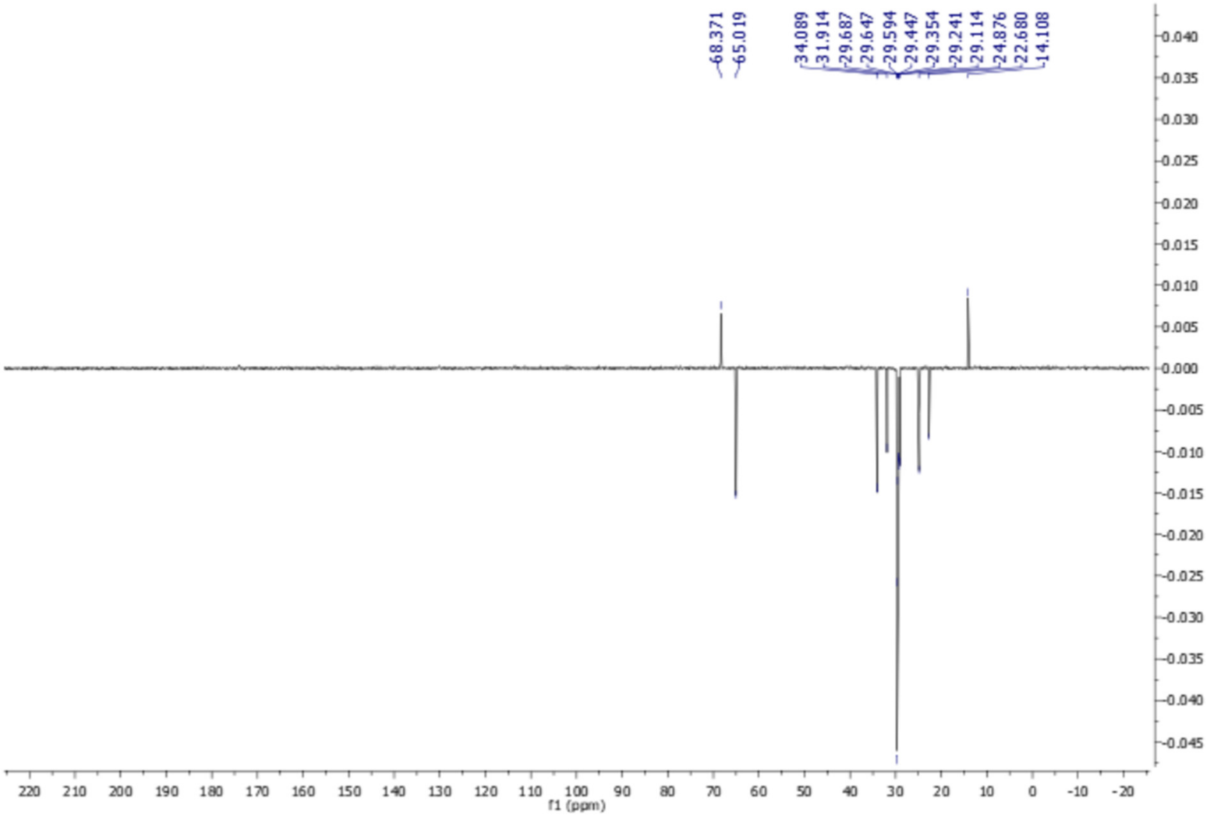

Figure S3. DEPT 135 spectrum of 1,3-distearoylglycerol (**3a**)

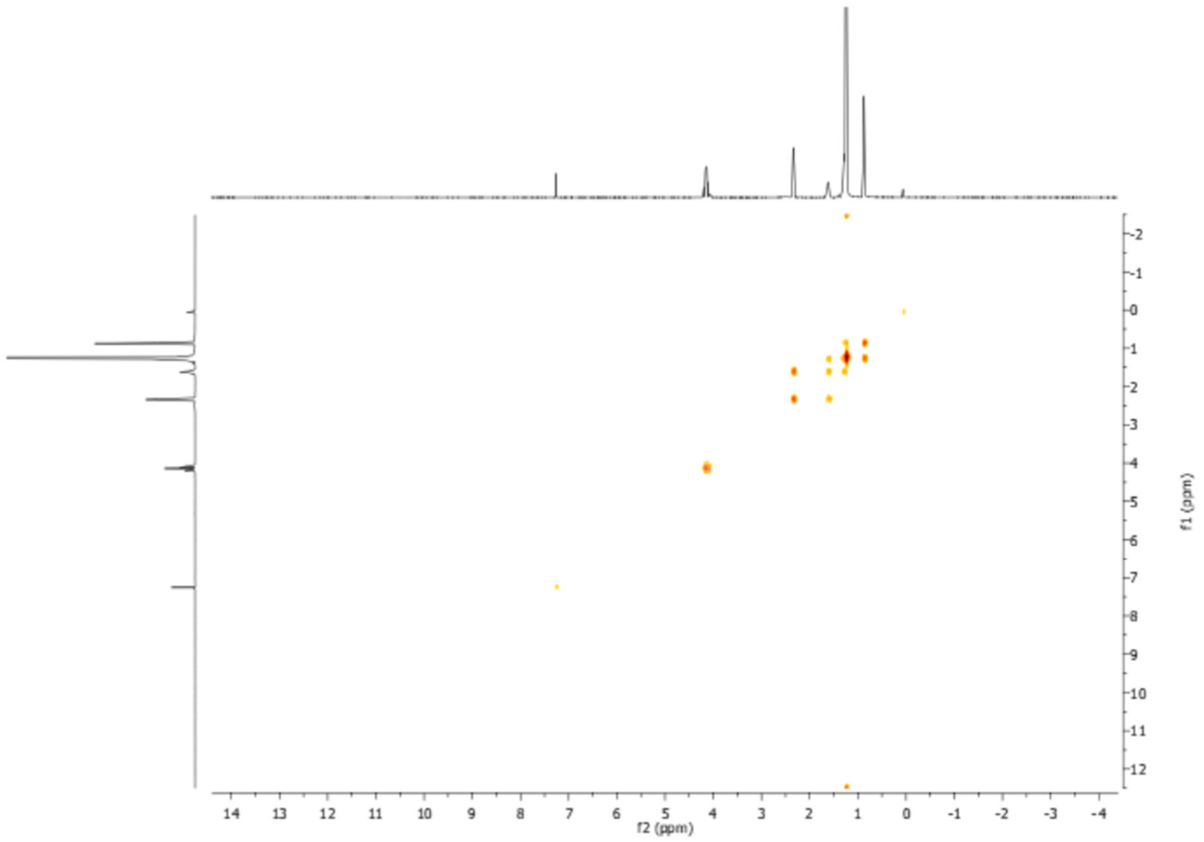

Figure S4. COSY spectrum of 1,3-distearoylglycerol (**3a**)

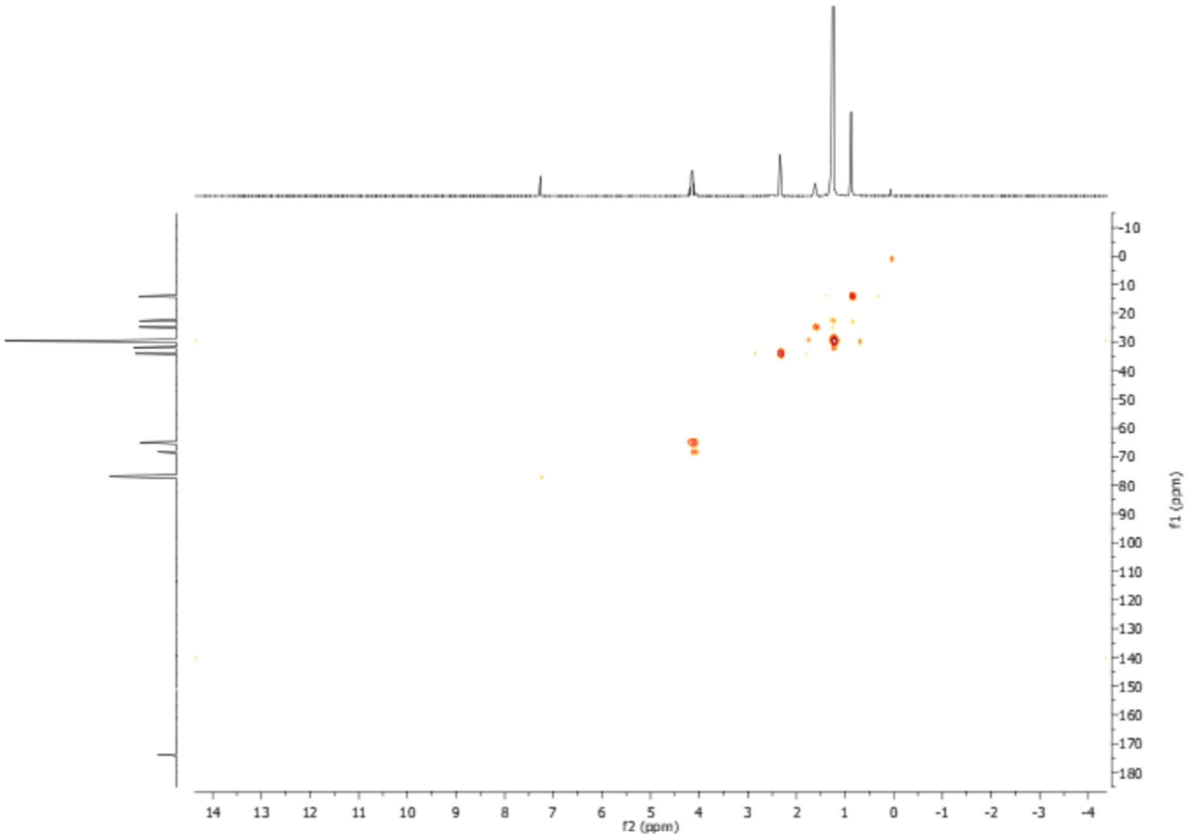

Figure S5. HMQC spectrum of 1,3-distearoylglycerol (**3a**)

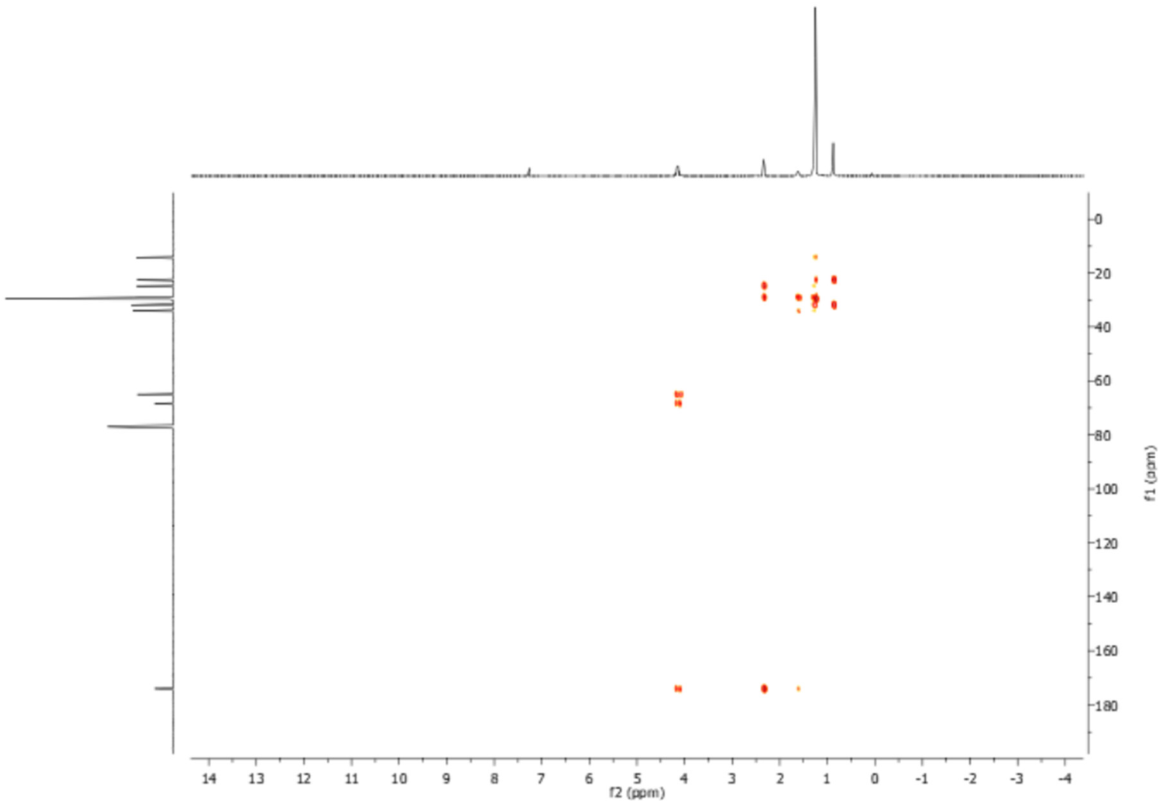

Figure S6. HMBC spectrum of 1,3-distearoylglycerol (**3a**)

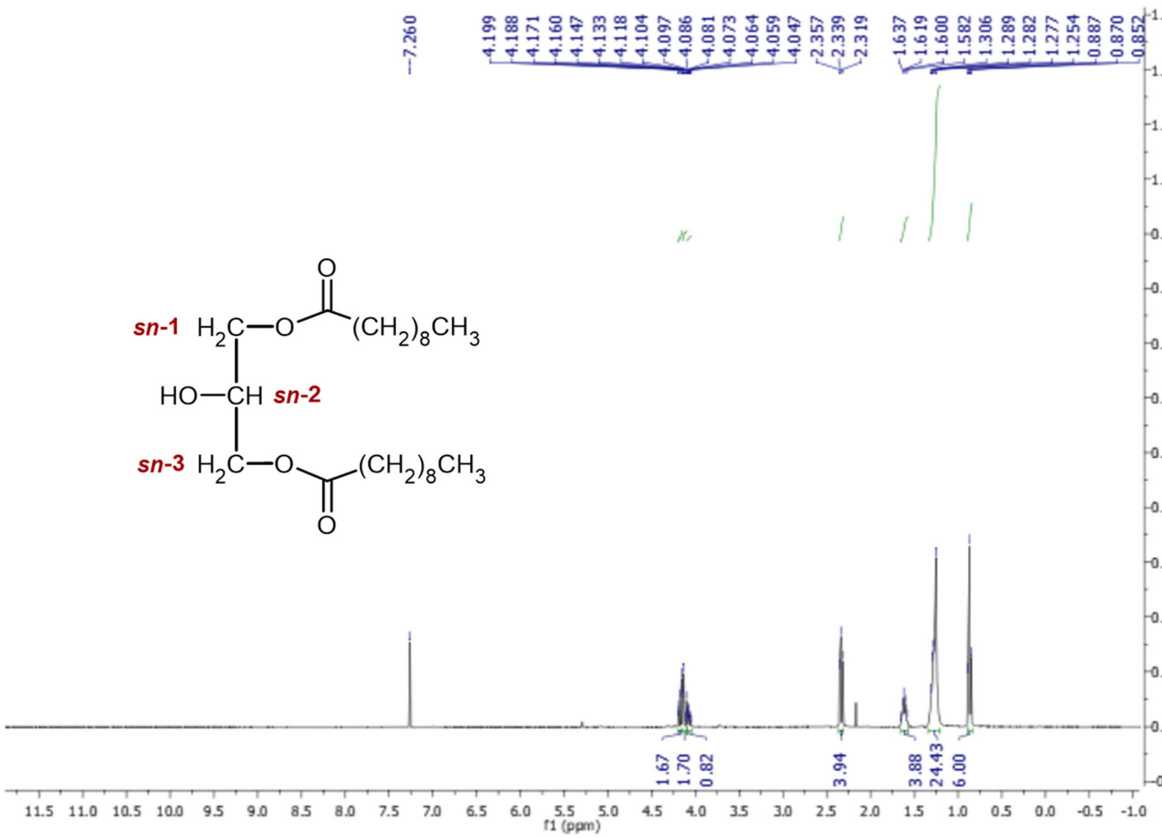

Figure S7. ^{1}H NMR spectrum of 1,3-didecanoylglycerol (**3b**)

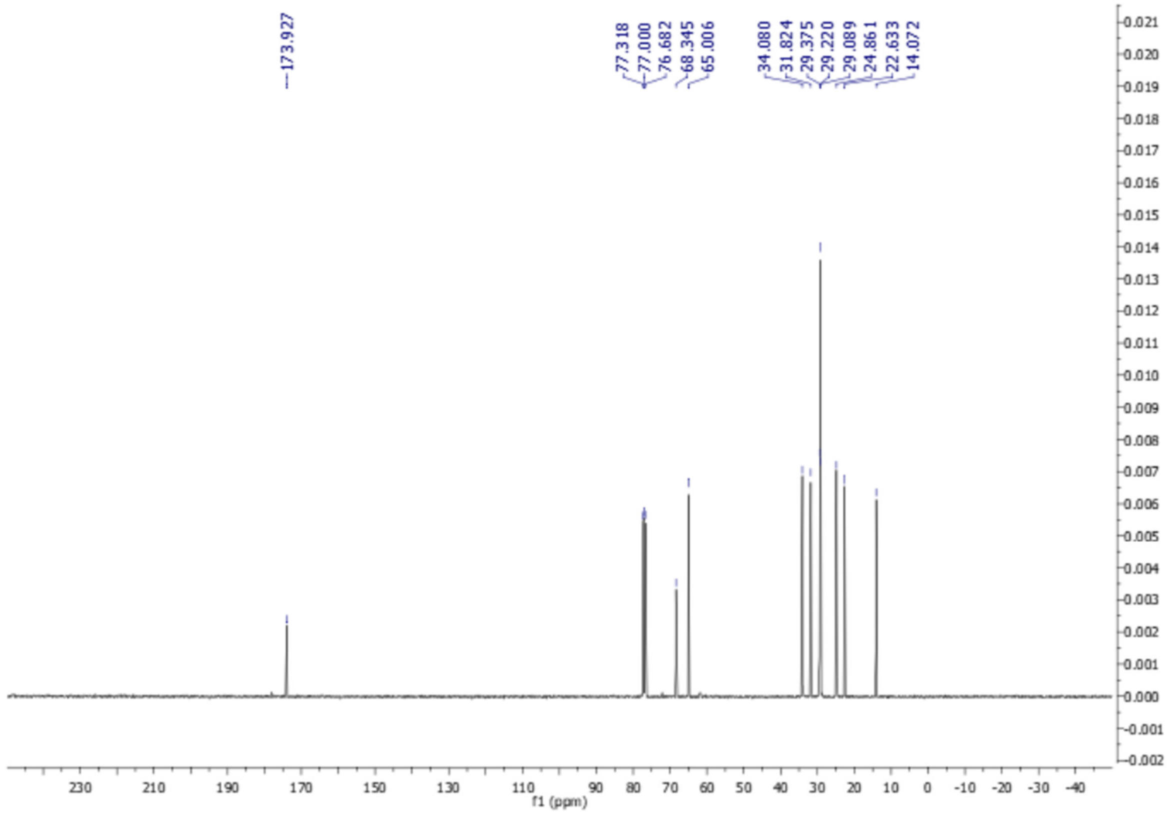

Figure S8. ^{13}C NMR spectrum of 1,3-didecanoylglycerol (**3b**)

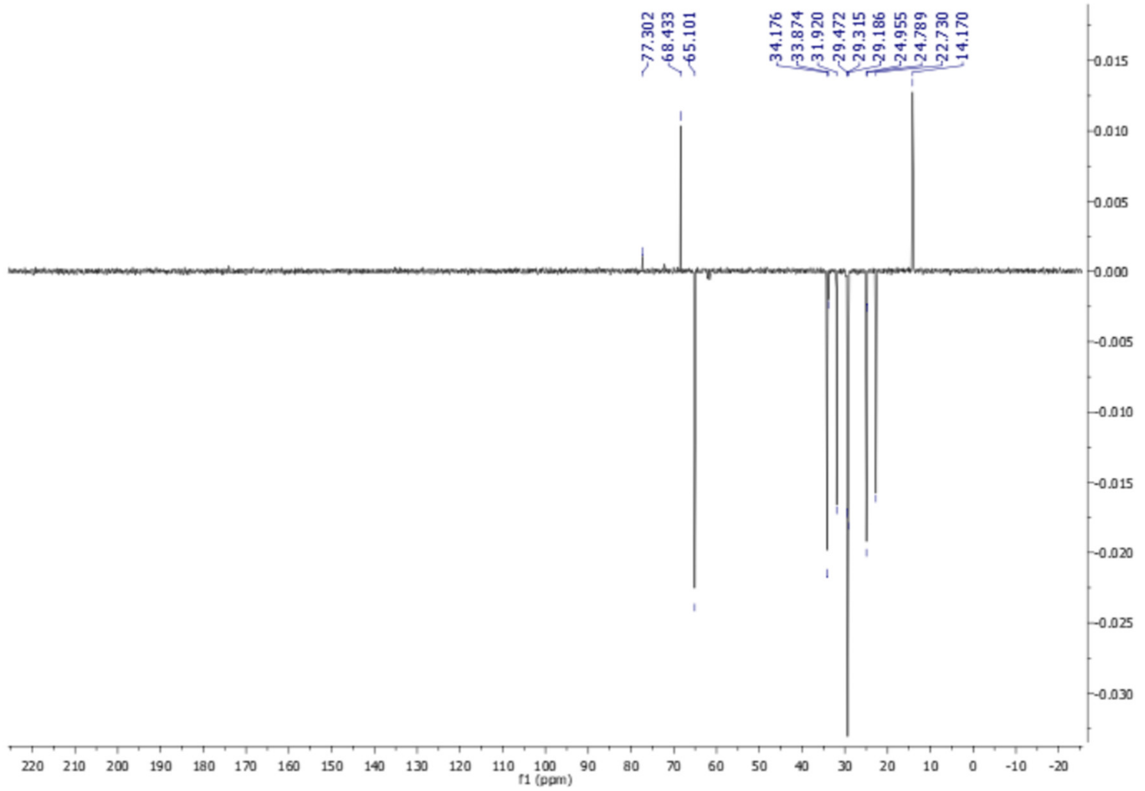

Figure S9. DEPT 135 spectrum of 1,3-didecanoylglycerol (**3b**)

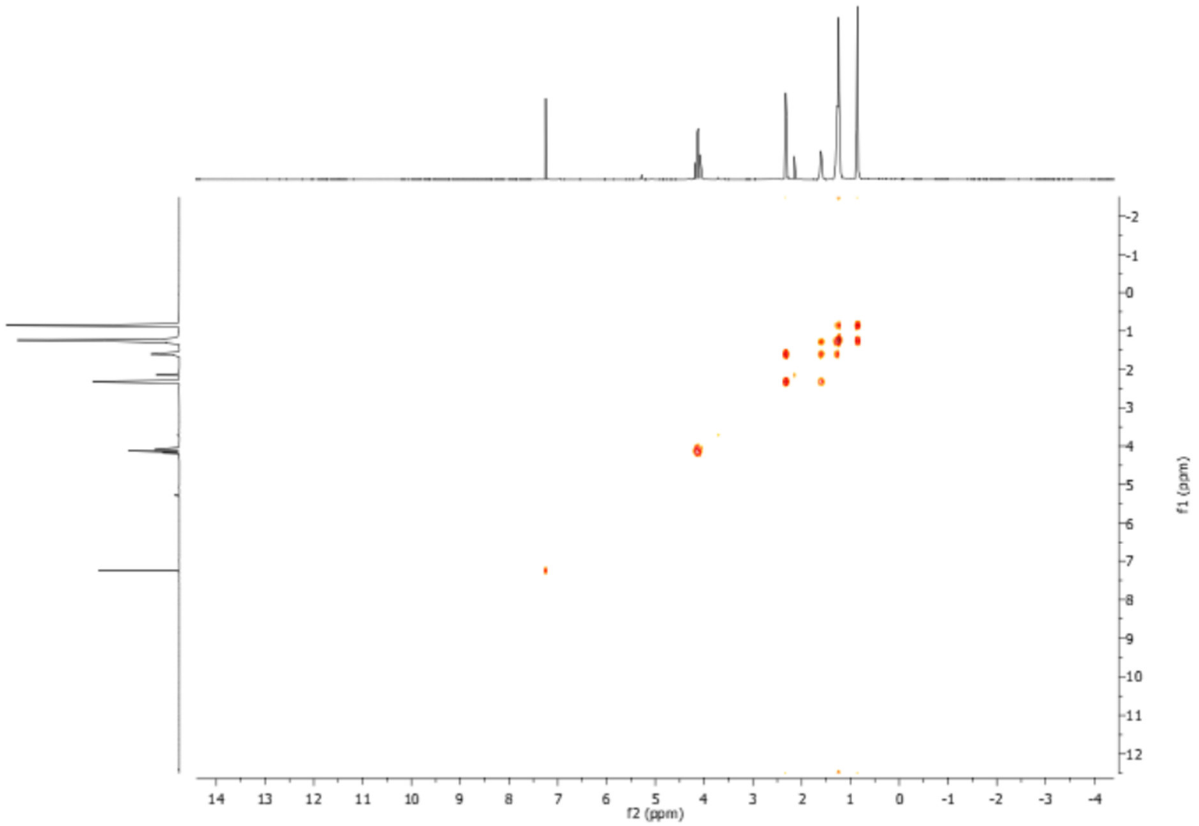

Figure S10. COSY spectrum of 1,3-didecanoylglycerol (**3b**)

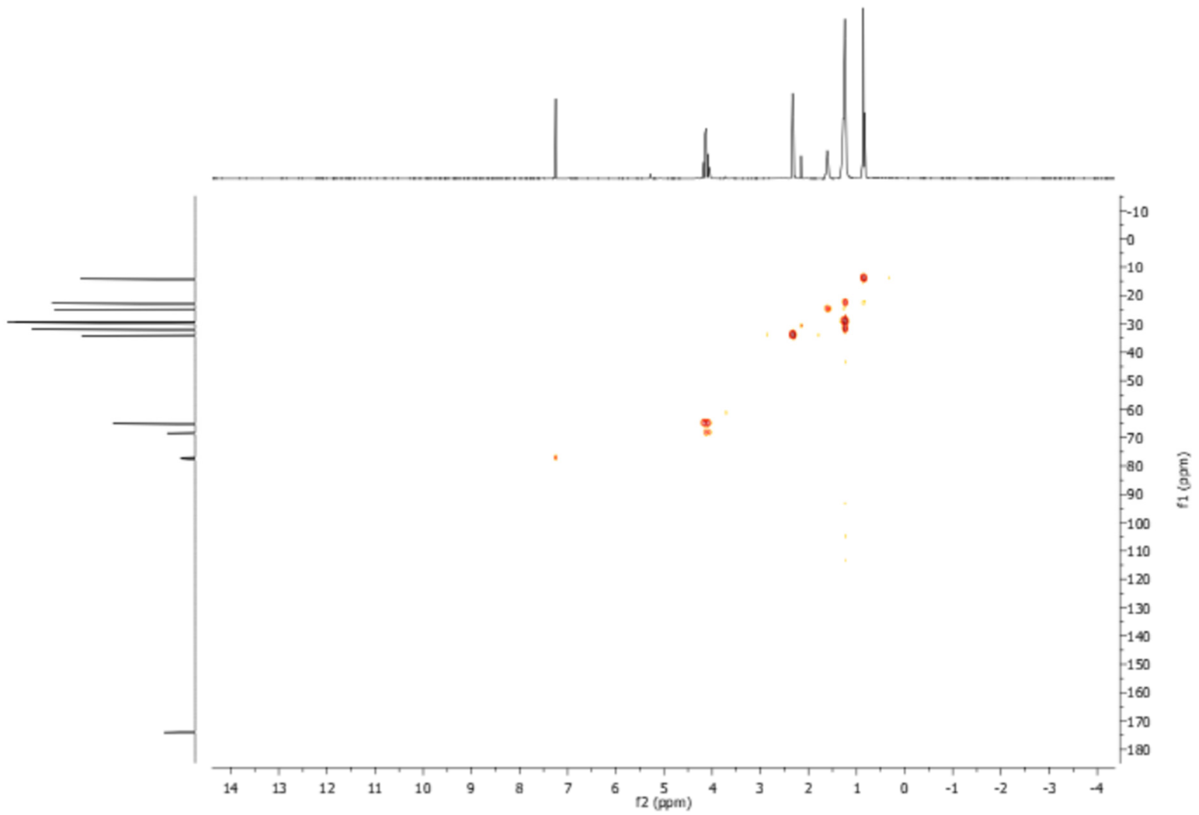

Figure S11. HMQC spectrum of 1,3-didecanoylglycerol (**3b**)

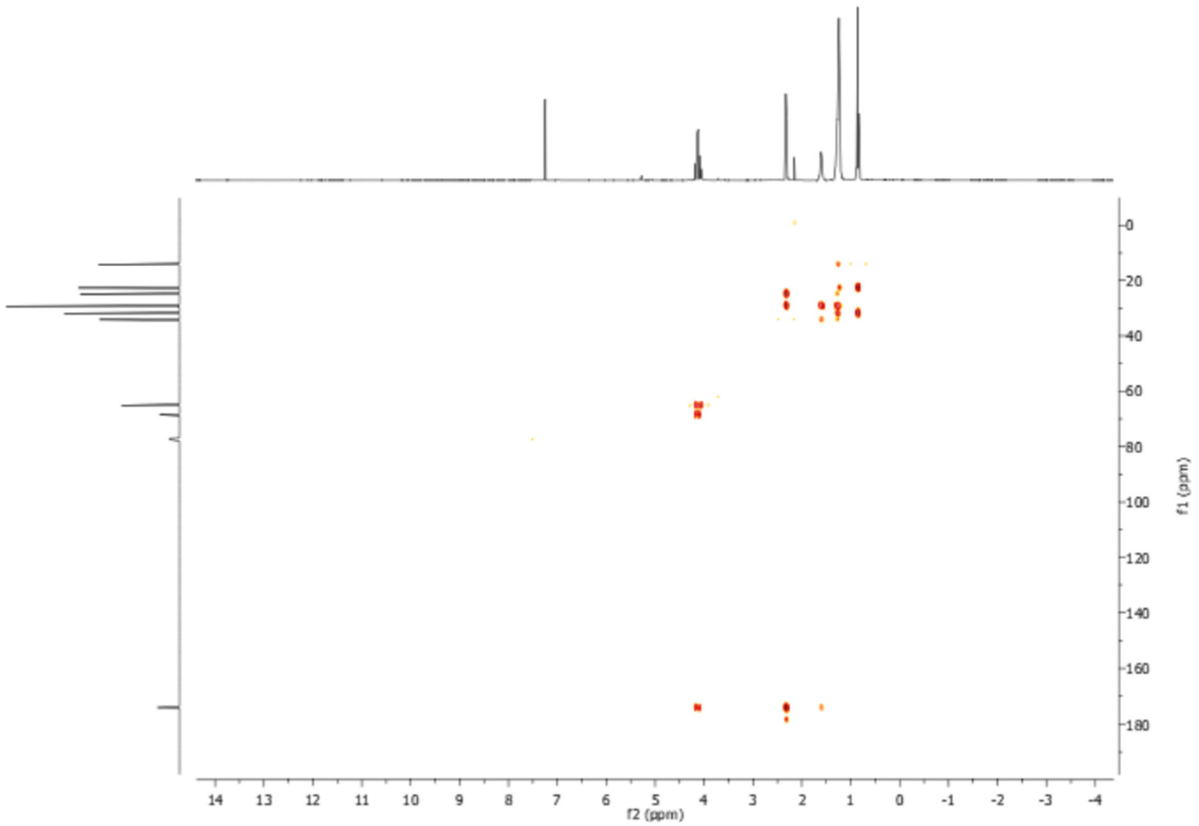

Figure S12. HMBC spectrum of 1,3-didecanoylglycerol (**3b**)

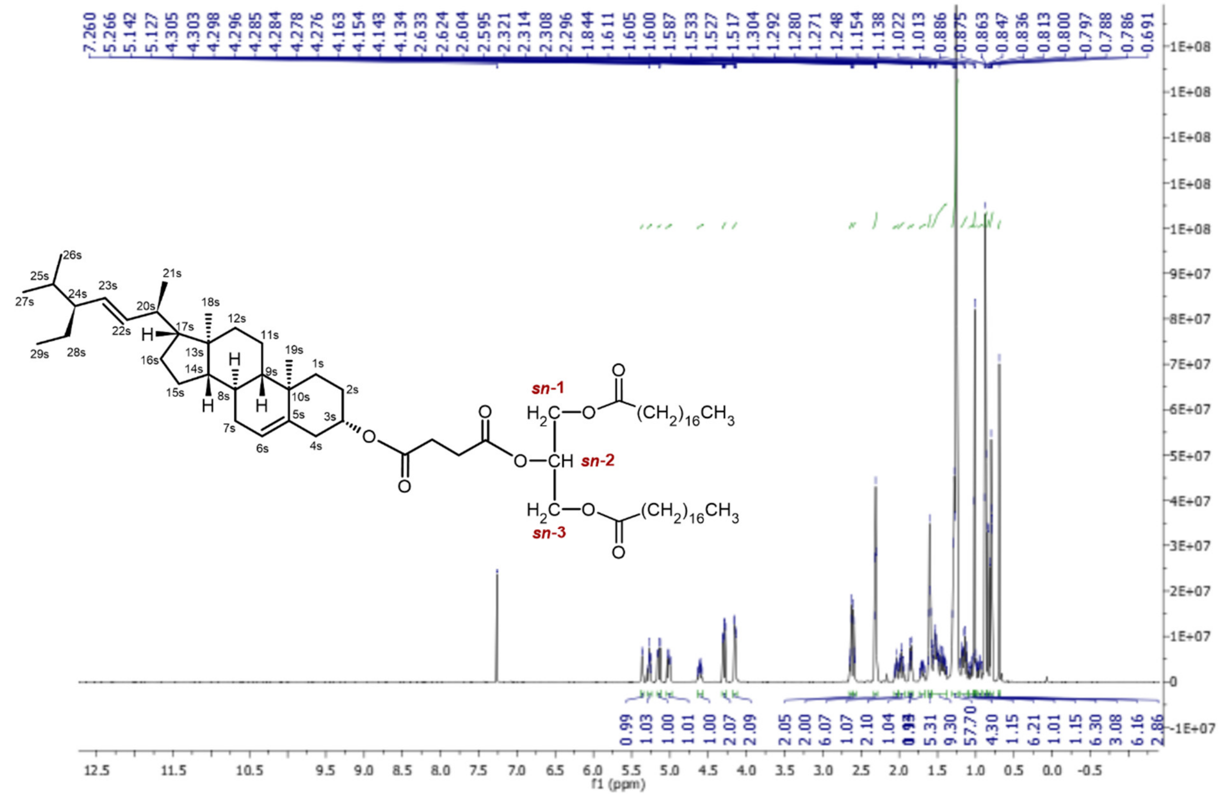

Figure S13. ^{1}H NMR spectrum of 1,3-distearoyl-2-stigmasterylsuccinoylglycerol (**4a**)

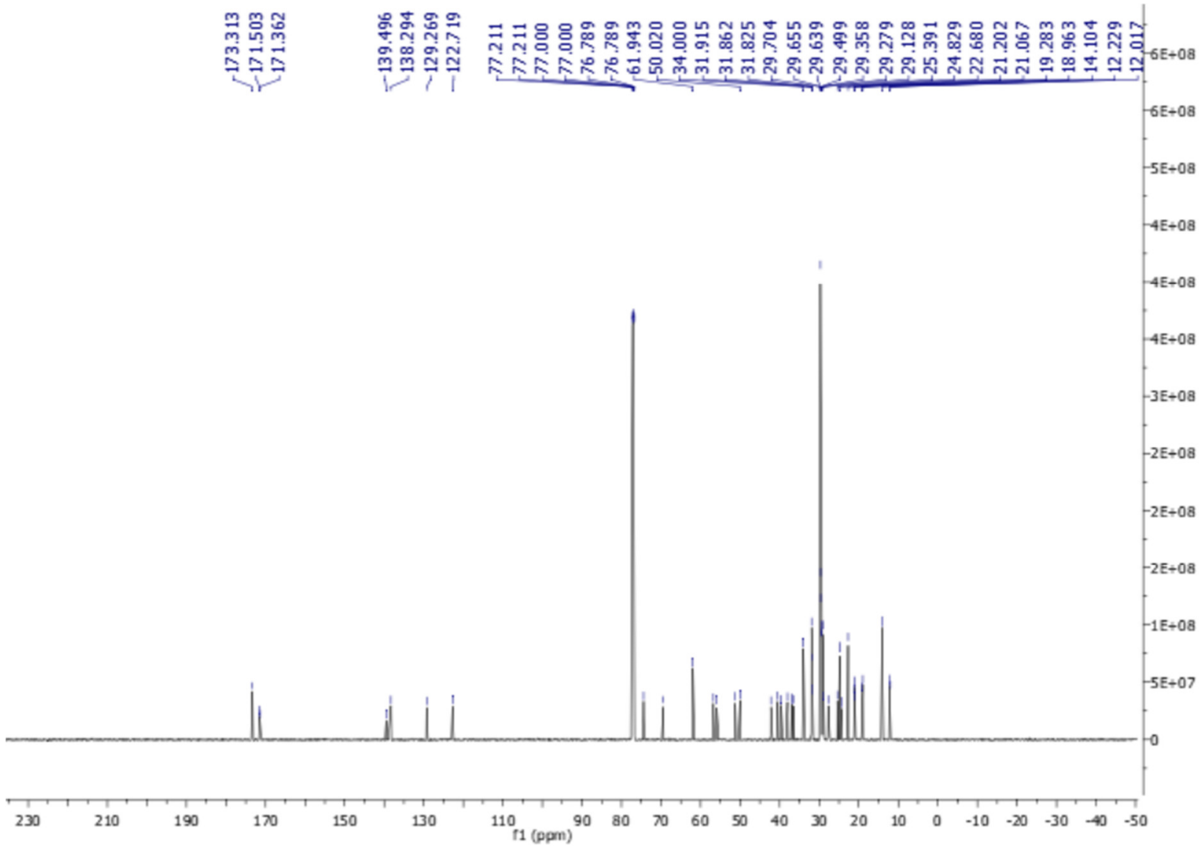

Figure S14. ^{13}C NMR spectrum of 1,3-distearoyl-2-stigmasterylsuccinoylglycerol (**4a**)

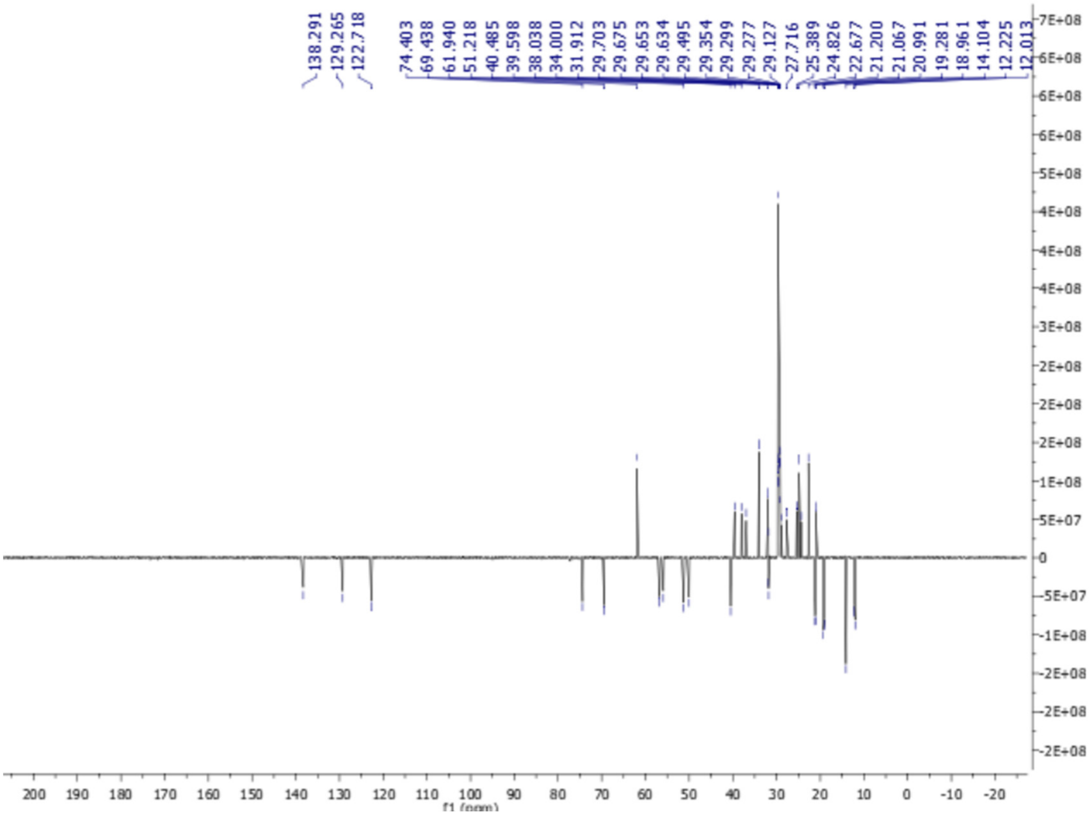

Figure S15. DEPT 135 spectrum of 1,3-distearoyl-2-stigmasterylsuccinoylglycerol (**4a**)

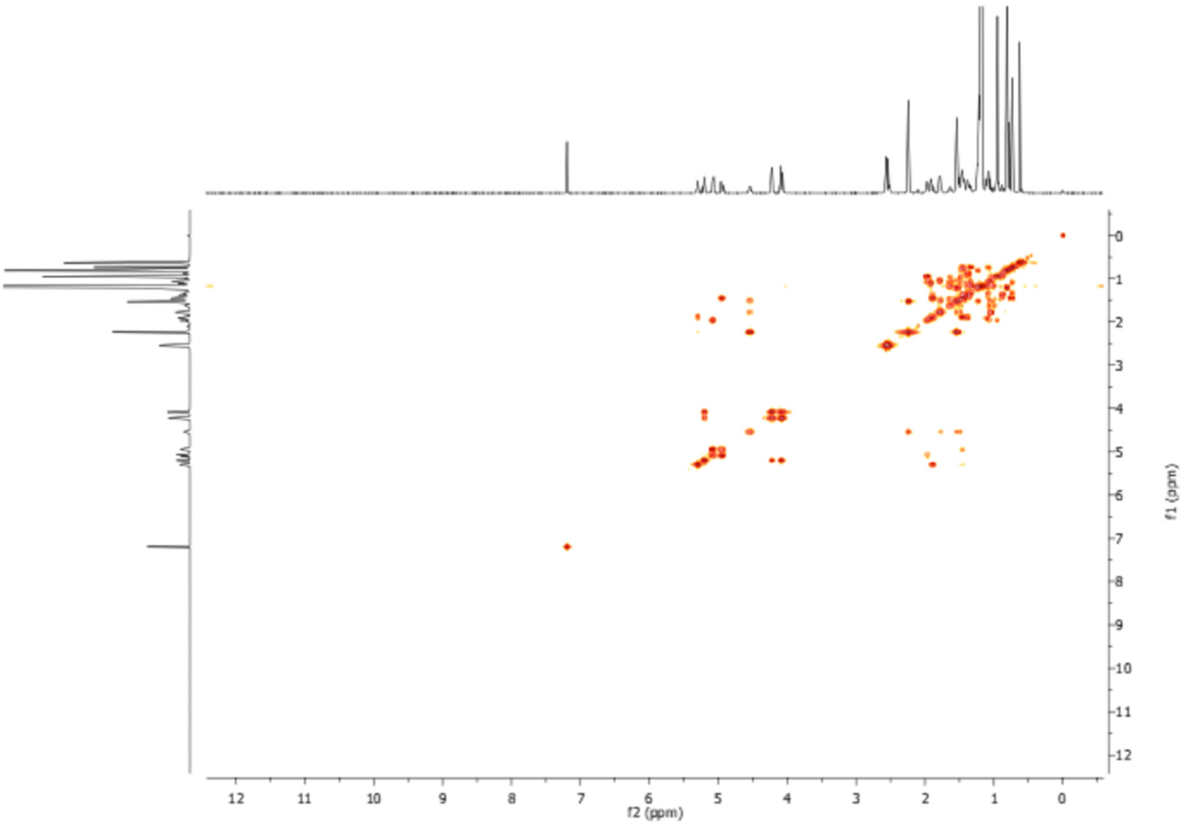

Figure S16. COSY spectrum of 1,3-distearoyl-2-stigmasterylsuccinoylglycerol (**4a**)

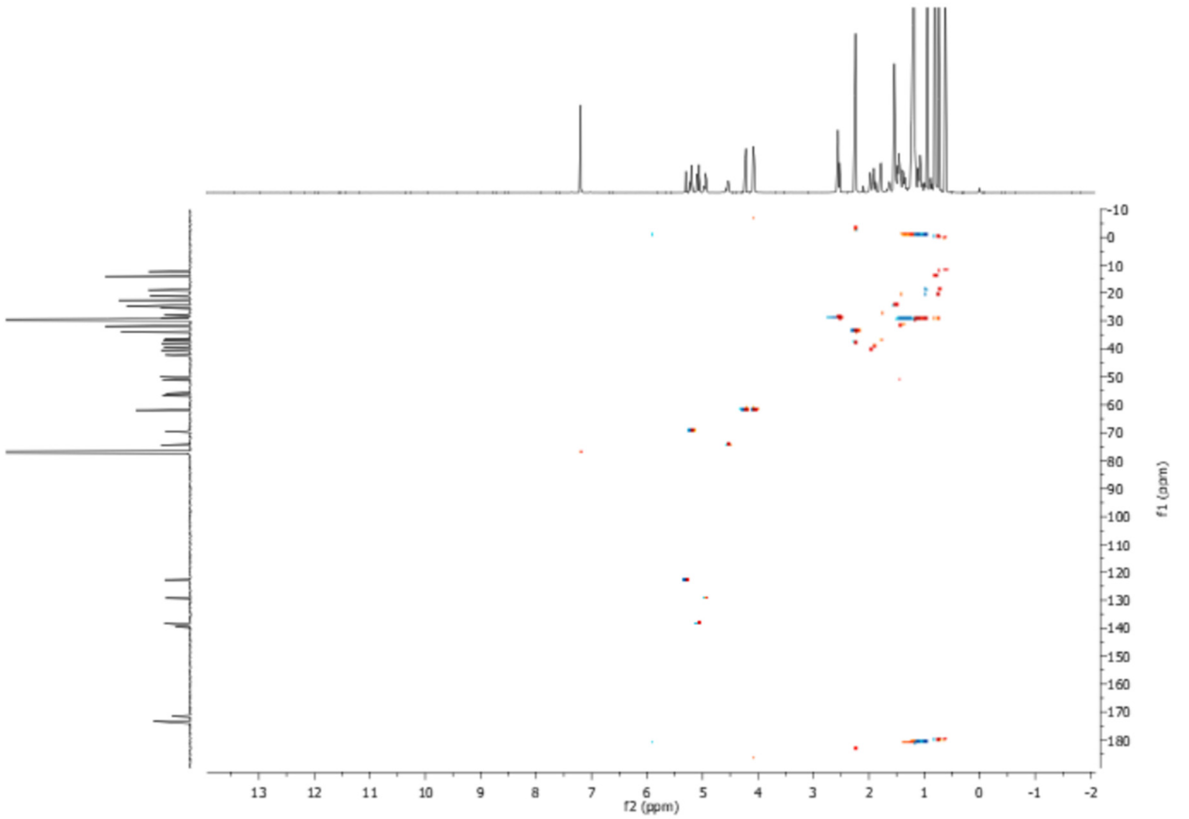

Figure S17. HSQC spectrum of 1,3-distearoyl-2-stigmasterylsuccinoylglycerol (**4a**)

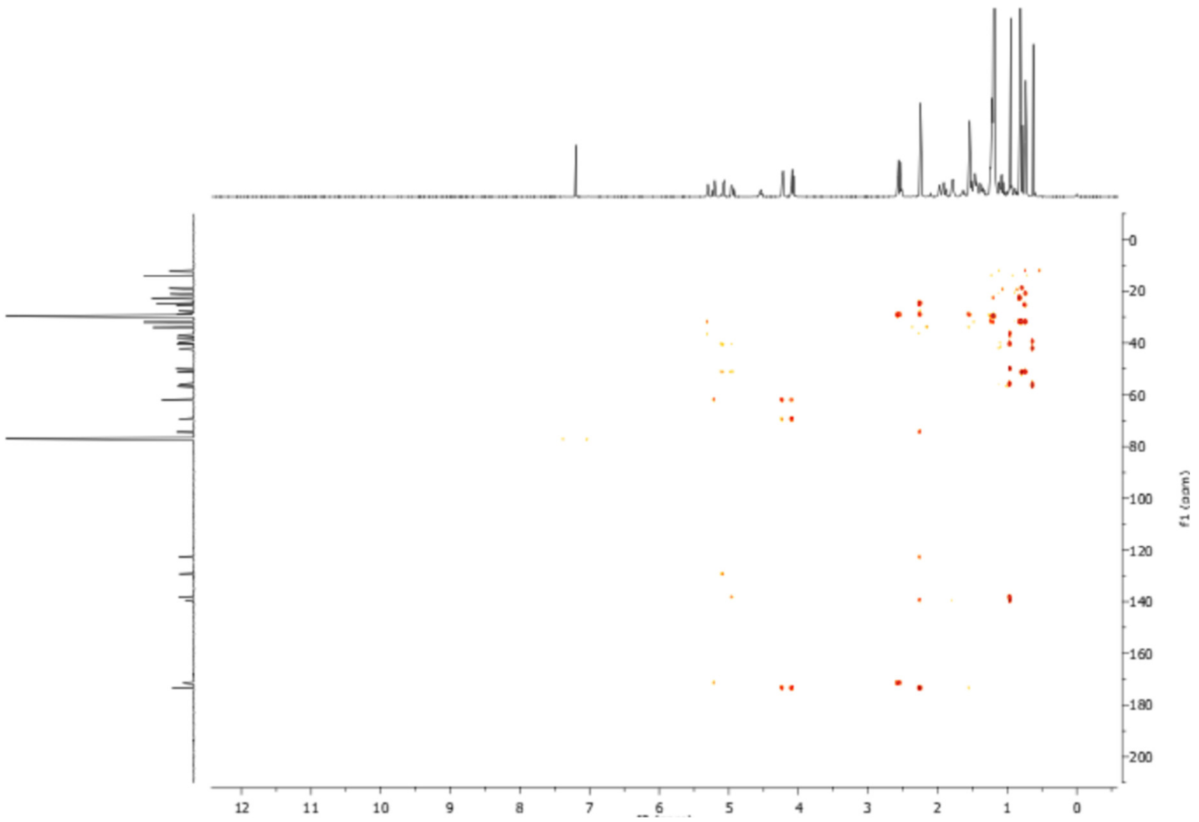

Figure S18. HMBC spectrum of 1,3-distearoyl-2-stigmasterylsuccinoylglycerol (**4a**)

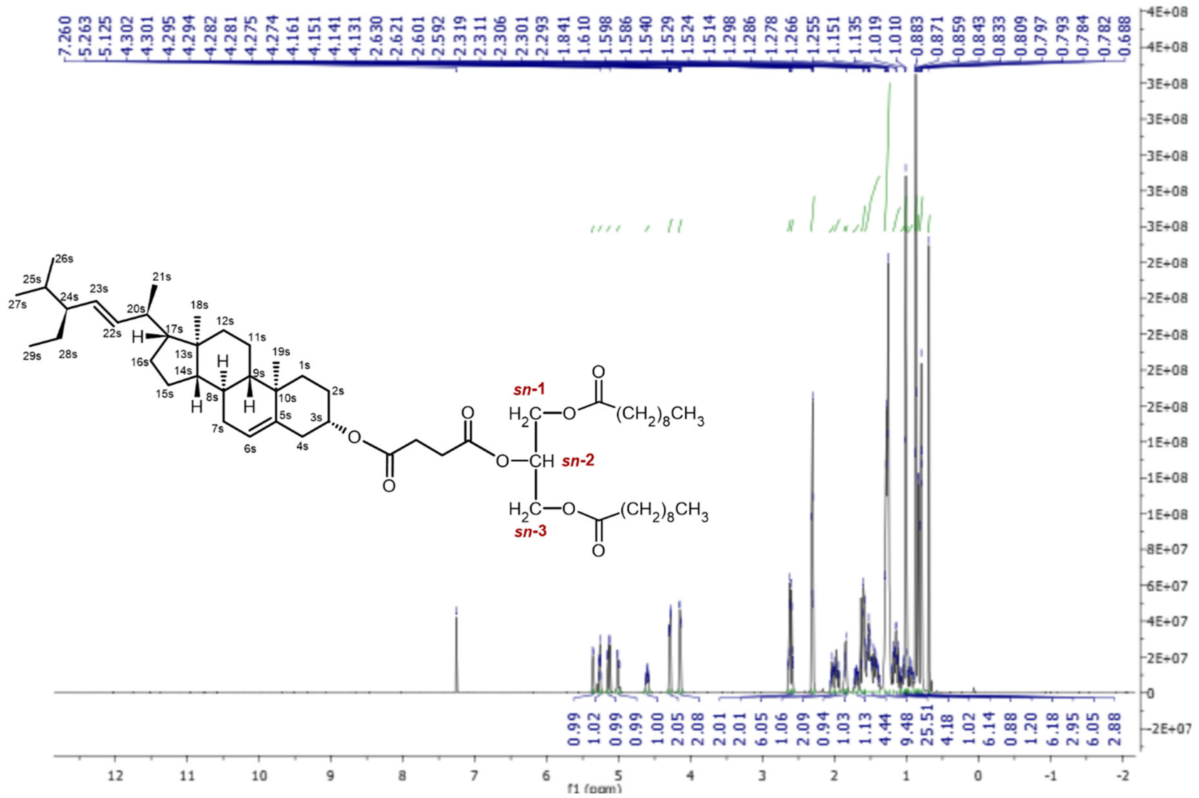

Figure S19. ^{1}H NMR spectrum of 1,3-didecanoyl-2-stigmasterylsuccinoylglycerol (**4b**)

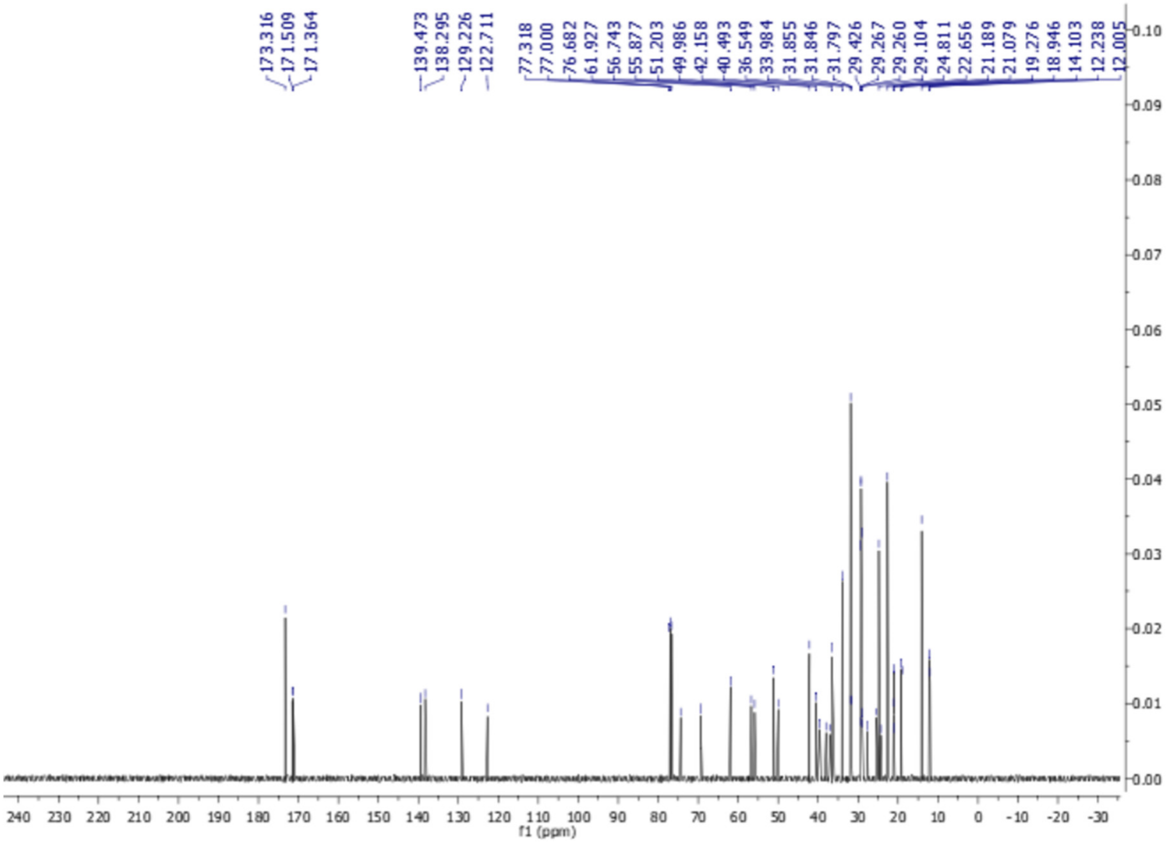

Figure S20. ^{13}C NMR spectrum of 1,3-didecanoyl-2-stigmasterylsuccinoylglycerol (**4b**)

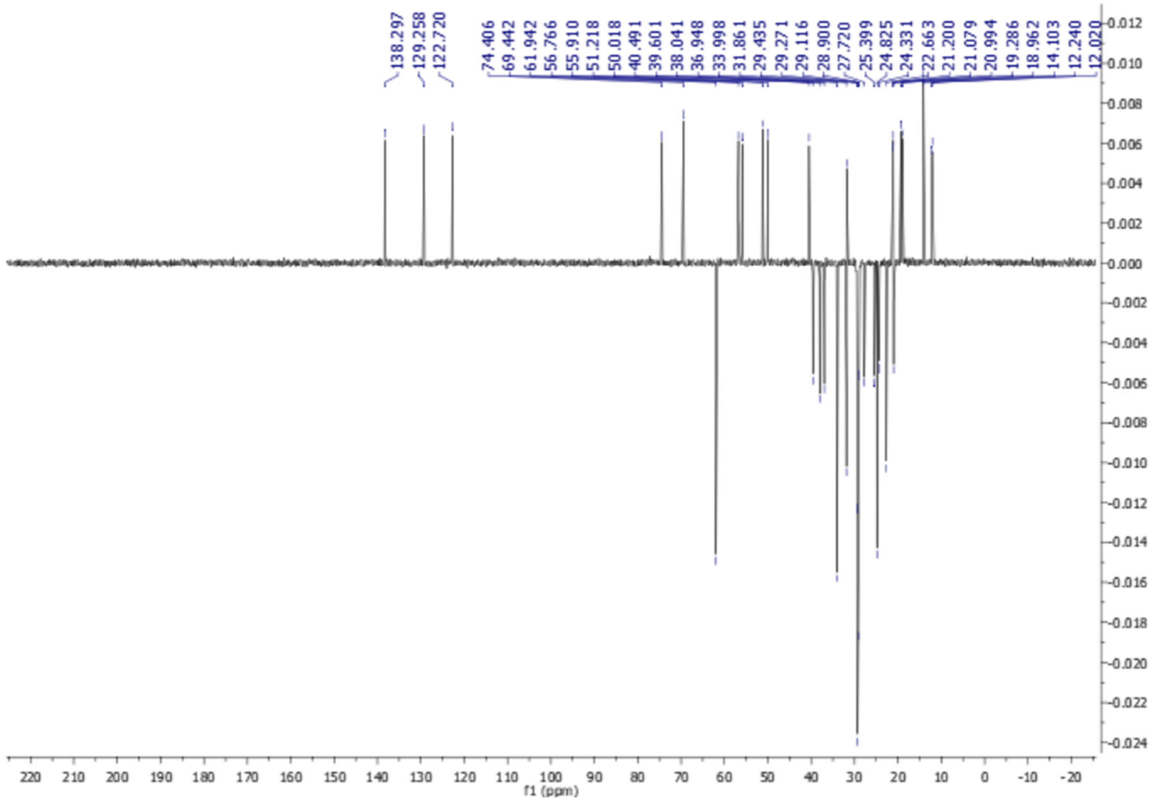

Figure S21. DEPT 135 spectrum of 1,3-didecanoyl-2-stigmasterylsuccinoylglycerol (**4b**)

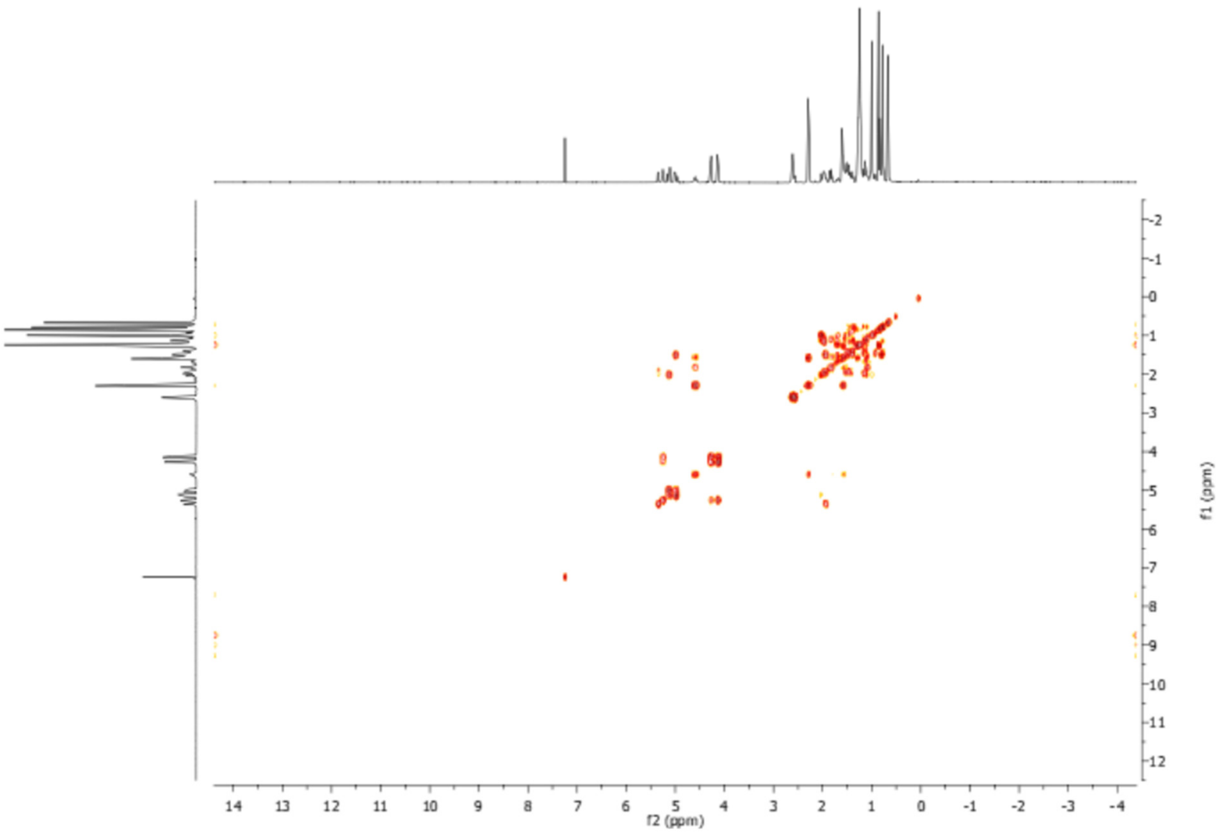

Figure S22. COSY spectrum of 1,3-didecanoyl-2-stigmasterylsuccinoylglycerol (**4b**)

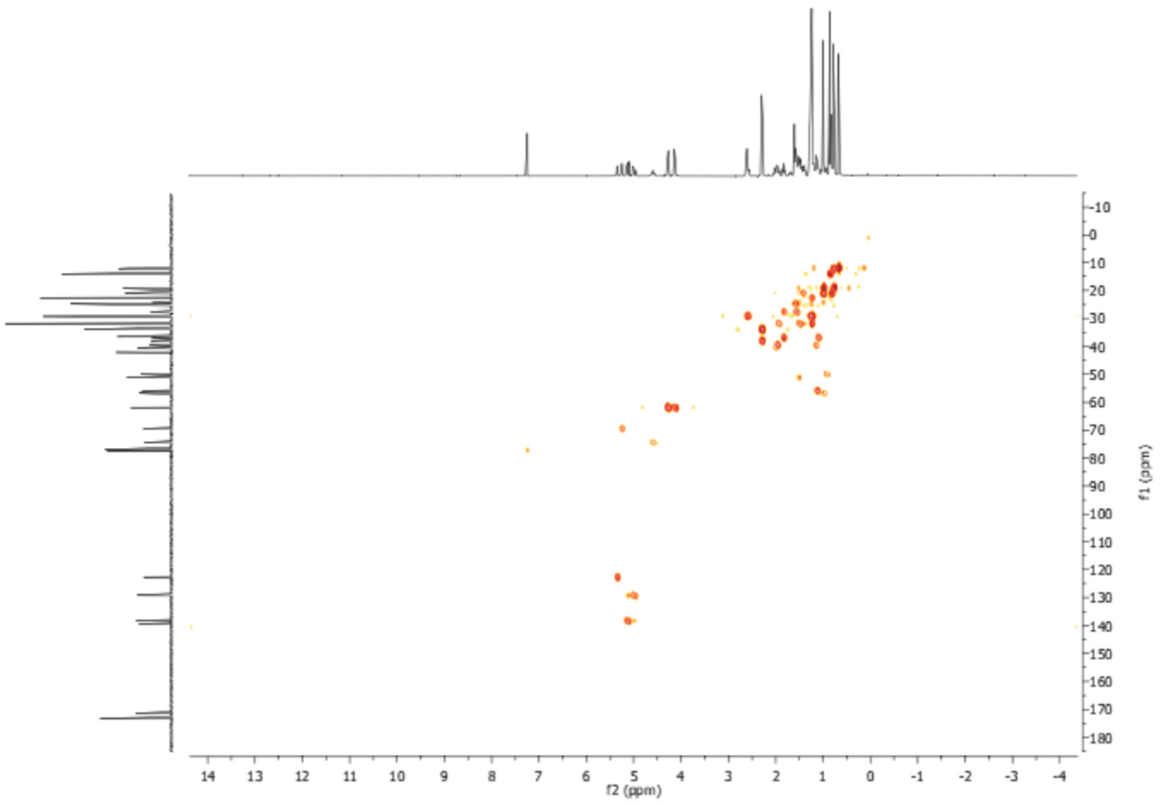

Figure S23. HMQC spectrum of 1,3-didecanoyl-2-stigmasterylsuccinoylglycerol (**4b**)

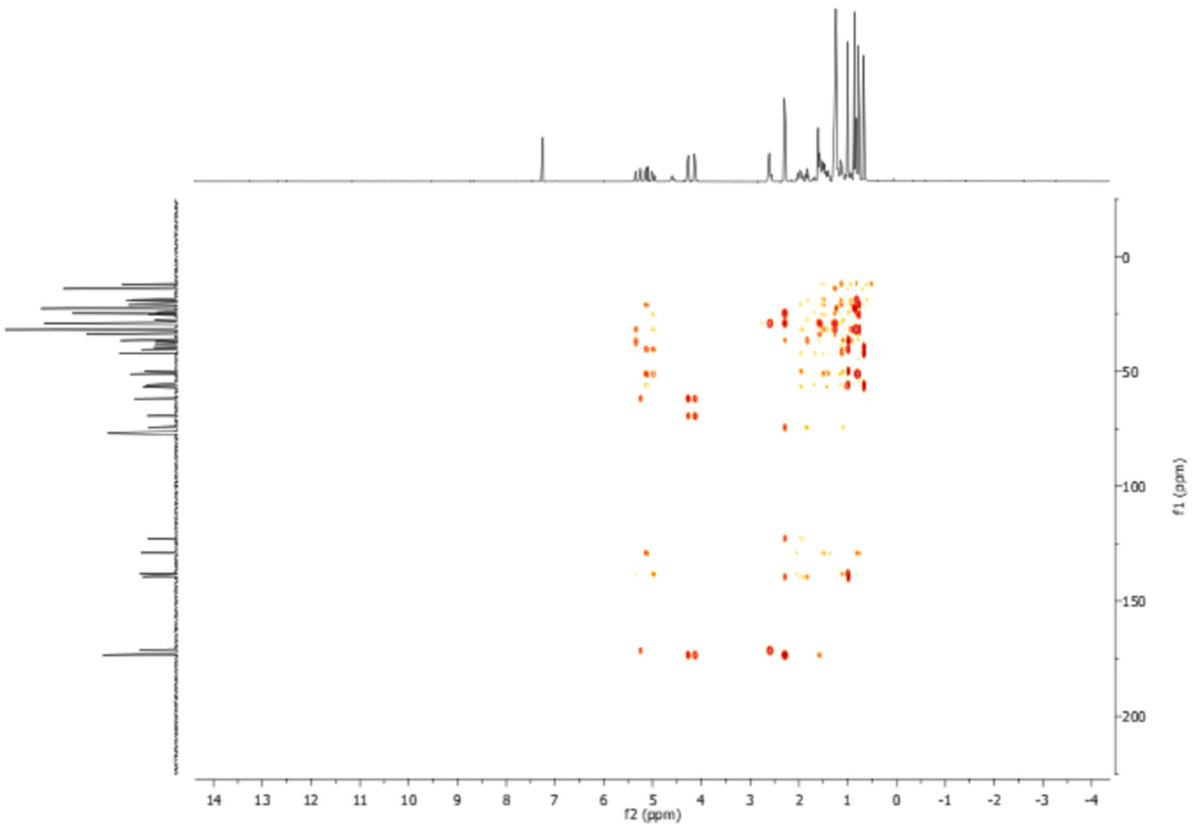

Figure S24. HMBC spectrum of 1,3-didecanoyl-2-stigmasterylsuccinoylglycerol (**4b**)

Table S1 Temperature dependence of average polydispersity index (PDI) for control (pure DPPC) and systems containing mixtures 1,3-didecanoyl-2-stigmasterylsuccinoylglycerol (**4b**) and DPPC at different molar ratio of ($n_{acylglycerol}/n_{DPPC}$): 0.1, 0.025, and 0.0125. Values are presented as mean ± standard deviation (*n* = 3).

Temperature [℃]	**Average polydispersity index (PDI)**			
	DPPC	0.1	0.025	0.0125
25	**0.116** ± 0.005	**0.080** ± 0.004	**0.113** ± 0.005	**0.100** ± 0.014
30	**0.120** ± 0.007	**0.049** ± 0.008	**0.088** ± 0.025	**0.096** ± 0.001
35	**0.125** ± 0.003	**0.108** ± 0.007	**0.083** ± 0.009	**0.091** ± 0.011
37	**0.117** ± 0.005	**0.067**± 0.006	**0.082**± 0.016	**0.089** ± 0.016
39	**0.100** ± 0.002	**0.111** ± 0.004	**0.107** ± 0.004	**0.076** ± 0.021
41	**0.098** ± 0.014	**0.067** ± 0.007	**0.110** ± 0.008	**0.097** ± 0.006
42	**0.115** ± 0.005	**0.097** ± 0.010	**0.123** ± 0.004	**0.125** ± 0.005
43	**0.056** ± 0.006	**0.079** ± 0.007	**0.123** ± 0.019	**0.108** ± 0.015
45	**0.096** ± 0.018	**0.080** ± 0.016	**0.102** ± 0.014	**0.066** ± 0.032
50	**0.120** ± 0.009	**0.081** ± 0.015	**0.121** ± 0.003	**0.081** ± 0.016
55	**0.055** ± 0.046	**0.117** ± 0.020	**0.134** ± 0.005	**0.037** ± 0.028
60	**0.112** ± 0.008	**0.070** ± 0.001	**0.127** ± 0.008	**0.111** ± 0.014

Table S2. Temperature dependence of the average polydispersity index (PDI) for pure DPPC and 1,3-distearoyl-2-stigmasterylsuccinoylglycerol (**4a**)/DPPC mixtures at molar ratio of acylglycerol to DPPC ($n_{acylglycerol}/n_{DPPC}$): 0.1, 0.025, and 0.0125. Values are presented as mean ± standard deviation (*n* = 3).

Temperature [℃]	**Average polydispersity index (PDI)**			
	DPPC	0.1	0.025	0.0125
25	**0.147** ± 0.025	**0.186** ± 0.005	**0.109** ± 0.003	**0.098** ± 0.016
30	**0.119** ± 0.007	**0.152** ± 0.001	**0.112** ± 0.004	**0.096** ± 0.006
35	**0.130** ± 0.001	**0.128** ± 0.017	**0.118** ± 0.004	**0.108** ± 0.015
37	**0.151** ± 0.012	**0.126** ± 0.009	**0.112** ± 0.024	**0.065** ± 0.014
39	**0.129** ± 0.002	**0.115** ± 0.005	**0.096** ± 0.010	**0.071** ± 0.011
41	**0.138** ± 0.007	**0.106** ± 0.024	**0.109** ± 0.009	**0.078** ± 0.017
42	**0.121** ± 0.004	**0.093** ± 0.006	**0.101** ± 0.030	**0.097** ± 0.003
43	**0.113** ± 0.006	**0.050** ± 0.018	**0.117** ± 0.030	**0.100** ± 0.003
45	**0.145** ± 0.004	**0.076** ± 0.010	**0.081** ± 0.004	**0.088** ± 0.005
50	**0.143** ± 0.006	**0.065** ± 0.008	**0.134** ± 0.001	**0.122** ± 0.021
55	**0.172** ± 0.004	**0.064** ± 0.023	**0.122** ± 0.021	**0.095** ± 0.009
60	**0.146** ± 0.002	**0.073** ± 0.012	**0.120** ± 0.008	**0.093** ± 0.006

Table S3. Maxima of asymmetric and symmetric methylene (CH2) stretching vibration bands for pure DPPC and 1,3-didecanoyl-2-stigmasterylsuccinoylglycerol (**4b**)/DPPC of acylglycerol to DPPC ($n_{acylglycerol}/n_{DPPC}$): 0.01 and 0.025 molar ratios at temperatures

Temperature [°C]	Wavenumbers [cm $^{-1}$]					
	DPPC		0.01		0.025	
	$v_{as}(CH_2)$	$v_s(CH_2)$	$v_{as}(CH_2)$	$v_s(CH_2)$	$v_{as}(CH_2)$	$v_s(CH_2)$
25	2917.62	2849.63	2917.68	2849.97	2917.03	2849.64
30	2917.54	2849.86	2917.92	2850.05	2917.33	2849.80
35	2917.91	2850.11	2918.19	2850.29	2917.6	2849.93
37	2917.99	2850.15	2918.58	2850.53	2917.76	2849.99
39	2918.18	2850.25	2918.94	2850.71	2917.98	2850.11
41	2918.76	2850.60	2919.28	2850.78	2918.25	2850.53
42	2921.98	2852.76	2921.89	2852.82	2919.13	2851.06
45	2922.19	2853.01	2922.27	2853.11	2921.41	2852.30
50	2922.66	2853.28	2922.74	2853.51	2921.5	2852.33
55	2923.00	2853.55	2922.62	2853.35	2921.73	2852.49
60	2923.48	2854.00	2922.81	2853.54	2922.07	2852.71

Table S4. Maxima of asymmetric and symmetric methylene (CH2) stretching vibration bands for pure DPPC and 1,3-distearoyl-2-stigmasterylsuccinoylglycerol (**4a**)/DPPC mixtures of acylglycerol to DPPC ($n_{acylglycerol}/n_{DPPC}$): 0.01 and 0.025 molar ratios at temperatures.

Temperature [°C]	Wavenumbers [cm $^{-1}$]					
	DPPC		0.01		0.025	
	$v_{as}(CH_2)$	$v_s(CH_2)$	$v_{as}(CH_2)$	$v_s(CH_2)$	$v_{as}(CH_2)$	$v_s(CH_2)$
25	2917.29	2849.63	2917.45	2849.91	2916.95	2849.63
30	2917.59	2849.86	2917.90	2850.12	2917.36	2849.78
35	2917.72	2850.11	2918.15	2850.32	2917.55	2849.89
37	2917.78	2850.15	2918.25	2850.42	2917.69	2849.96
39	2917.85	2850.25	2918.34	2850.53	2917.96	2850.07
41	2918.45	2850.60	2919.16	2850.96	2918.38	2850.27
42	2921.75	2852.76	2921.65	2852.32	2919.10	2851.55
45	2922.11	2853.01	2922.06	2852.61	2921.29	2852.06
50	2922.46	2853.28	2922.42	2852.87	2921.72	2852.35
55	2922.71	2853.55	2922.77	2853.13	2922.03	2852.57
60	2922.98	2854.00	2923.06	2853.36	2922.32	2852.77
